# Determinants of Colorectal Cancer: An Integrative Immunometabolic Framework Linking Biomarkers, Therapy, and the Diet–Microbiota Axis

**DOI:** 10.3390/cells15121074

**Published:** 2026-06-13

**Authors:** Gianluca Aguiari, Nicoletta Bianchi, Ornella Franzese

**Affiliations:** 1 Department of Neuroscience and Rehabilitation, University of Ferrara, Via Fossato di Mortara 74, 44121 Ferrara, Italy; gianluca.aguiari@unife.it; 2 Department of Translational Medicine, University of Ferrara, Via Fossato di Mortara 70, 44121 Ferrara, Italy; 3 Department of Systems Medicine, University of Rome Tor Vergata, Via Montpellier 1, 00133 Rome, Italy; franzese@uniroma2.it

**Keywords:** colorectal cancer, metabolic biomarkers, cancer metabolism, tumor microenvironment, immunotherapy, microbiota, obesity, therapeutic resistance

## Abstract

**Highlights:**

**What are the main findings?**

- Colorectal cancer progression is driven by interconnected immunometabolic programs involving tumor metabolism, immune-cell function, obesity, and the diet microbiota axis rather than by isolated genetic alterations alone.
- Metabolic biomarkers and immunometabolic signatures emerging from glycolysis, mitochondrial metabolism, lipid and amino acid pathways, autophagy, and systemic metabolic factors may improve patient stratification and identify novel therapeutic vulnerabilities.

**What are the implications of the main findings?**

- An integrated immunometabolic framework may complement current molecular classifications and provide a more comprehensive understanding of colorectal cancer heterogeneity and treatment response.
- Targeting metabolic interactions within the tumor microenvironment, particularly in immunotherapy-resistant microsatellite-stable colorectal cancer, may enable the development of more effective precision medicine strategies.

**Abstract:**

Colorectal cancer (CRC) remains a leading cause of cancer-related morbidity and mortality, with substantial heterogeneity that is not fully explained by genetic alterations alone. Emerging evidence positions metabolic reprogramming as a central driver of tumor behavior, integrating glycolysis, mitochondrial function, lipid and amino acid metabolism, and autophagy into coordinated networks that extend beyond cancer cells to the tumor microenvironment. Tumor–immune metabolic competition and metabolite-mediated signaling shape immune responses, often promoting immunosuppression and resistance to immunotherapy, particularly in microsatellite-stable (MSS) CRC. Systemic factors, including obesity, insulin resistance, and the diet–microbiota axis, further modulate tumor metabolism and immune function, reinforcing disease progression. Metabolic biomarkers reflecting these multi-level interactions, spanning tumor-intrinsic pathways, immune contexture, and host metabolism, offer promising opportunities for improved patient stratification and therapeutic targeting, although clinical validation remains limited. Current treatments, including chemotherapy, targeted agents, and immune checkpoint inhibitors, are effective in selected subgroups but are constrained by resistance mechanisms. In this review, we propose an integrative immunometabolic framework in which tumor, immune, and systemic metabolic processes co-evolve, defining CRC progression and treatment response. Targeting this interconnected network through combinatorial and metabolism-oriented strategies may enable precision therapies, particularly for immunotherapy-resistant MSS CRC.

## 1. Introduction

Colorectal cancer (CRC) is one of the most commonly diagnosed malignancies, with approximately 2 million new cases worldwide in 2022, and remains the second leading cause of cancer-related mortality [1]. Although this trend has declined in recent decades likely owing to improvements in prevention, screening, diagnosis, and therapy [2,3], advanced disease still presents substantial therapeutic challenges. CRC is traditionally classified according to its genetic architecture [4,5,6,7]. However, this framework does not fully explain the marked heterogeneity observed among tumors sharing similar molecular alterations, particularly regarding growth dynamics, metastatic potential, immune interactions, and therapeutic response, suggesting that additional regulatory layers actively shape CRC biology beyond genetic alterations alone.

Metabolic organization has emerged as a major determinant of tumor behavior. CRC cells activate coordinated metabolic programs involving glycolysis, mitochondrial respiration, lipid remodeling, amino acid metabolism, and autophagy in response to environmental and therapeutic pressures. Rather than functioning as isolated pathways, these processes form interconnected metabolic states that influence proliferation, invasion, immune escape, and drug sensitivity. The metabolic adaptations extend beyond tumor cells into the tumor microenvironment (TME), where nutrient competition, metabolite exchange, and redox constraints shape stromal and immune cell function. This is particularly relevant in microsatellite-stable (MSS) CRC, in which immune checkpoint inhibitors (ICIs) show limited efficacy in most patients [8], suggesting that resistance may partly reflect immunometabolic constraints within the TME.

Current therapeutic strategies only partially capture this complexity. Chemotherapy, targeted therapies against Epidermal growth factor receptor (EGFR), Vascular endothelial growth factor (VEGF), and ICIs have improved outcomes in selected patient subgroups [9,10,11,12]. However, treatment selection still relies predominantly on molecular markers such as *Rat Sarcoma (RAS)/B Rapidly Accelerated Fibrosarcoma (BRAF)* mutations and mismatch repair status [4,6,13,14,15], which do not fully reflect the functional biological states governing treatment response and disease progression. This limitation is especially evident in MSS CRC, where reliable predictive biomarkers remain limited.

In addition, CRC development and progression are strongly influenced by systemic metabolic conditions. Obesity, insulin resistance, chronic inflammation, adipose tissue dysfunction, diet, and gut microbiota composition collectively modulate nutrient availability, endocrine signaling, immune tone, and inflammatory pathways. These interactions establish a dynamic diet/microbiota/immune axis that shapes tumor progression, therapeutic vulnerability, and disease evolution. These observations support a more integrated framework in which metabolic organization complements molecular classification and contributes to tumor behavior and immune contexture.

**Figure 1:**
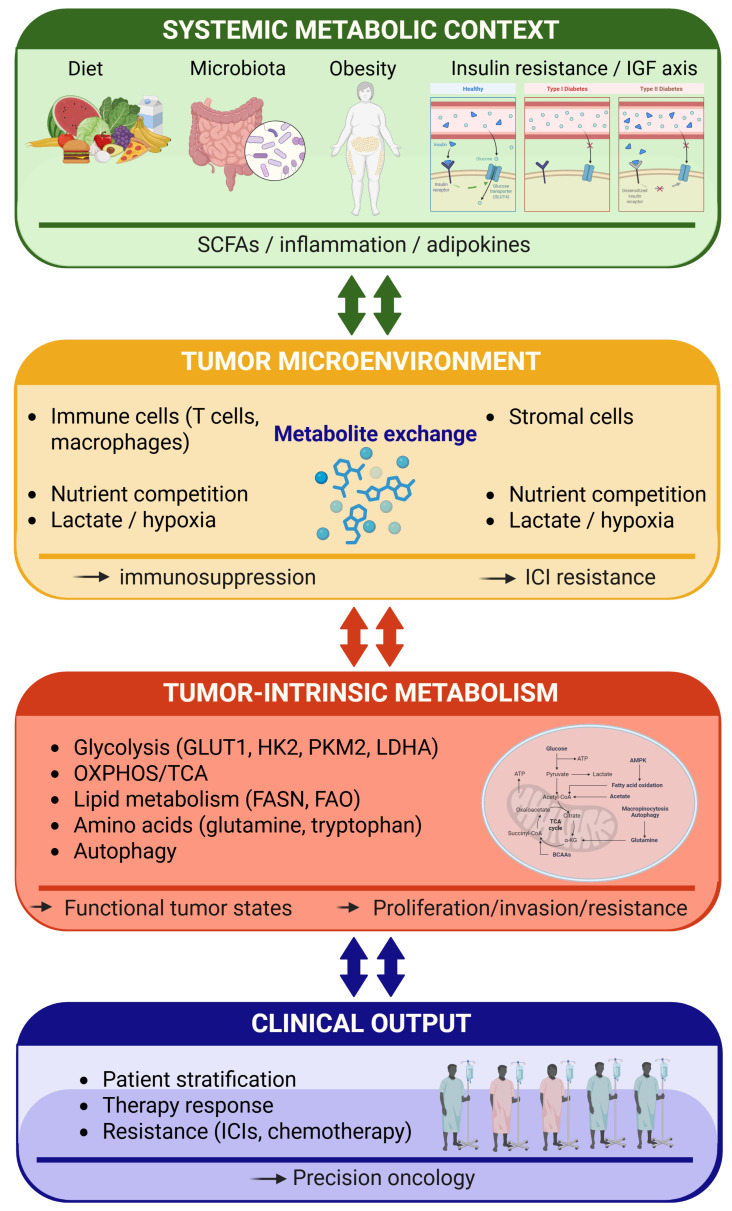
Multi-scale metabolic organization of CRC. Tumor-intrinsic metabolic programs interact with the TME and systemic metabolic factors, including diet, microbiota, obesity, and insulin resistance, to influence tumor behavior, immune regulation, therapeutic response, and clinical outcomes. Bidirectional interactions among these levels shape functional tumor states and contribute to immunosuppression, resistance to ICIs, and disease progression. Created with BioRender (https://app.biorender.com). Abbreviations: CRC, colorectal cancer; FAO, fatty acid oxidation; FASN, fatty acid synthase; GLUT1, glucose transporter 1; HK2, hexokinase 2; ICI, immune checkpoint inhibitor; IGF, insulin-like growth factor; LDHA, lactate dehydrogenase A; OXPHOS, oxidative phosphorylation; PKM2, pyruvate kinase M2; SCFAs, short-chain fatty acids; TCA, tricarboxylic acid cycle.

This review aims to examine metabolic pathways as interconnected components of CRC biology with potential translational value as prognostic and predictive biomarkers. We discuss how glycolysis, mitochondrial metabolism, lipid and amino acid utilization, autophagy, and metabolic signaling interact with the TME and systemic metabolic conditions to influence tumor progression, immune responses, and therapeutic resistance. By integrating tumor-intrinsic, microenvironmental, and systemic determinants, we propose an immunometabolic framework that may support improved patient stratification and the development of more effective personalized therapeutic strategies. This conceptual framework is described in Figure 1.

For this review, original research articles and reviews published during the past ten years were systematically selected, with emphasis on the most relevant contributions to the field. Earlier studies were included when necessary to support and contextualize the discussion.

## 2. Metabolic Biomarkers in CRC

Significant advances have improved overall survival (OS) in patients with metastatic CRC (mCRC); however, therapeutic resistance remains a pervasive challenge. This phenomenon arises through multiple, often overlapping mechanisms, including acquired genetic mutations, epigenetic remodeling, adaptive changes within the TME, and rewiring of key signaling pathways, all of which constrain durable therapeutic benefit and drive disease progression [16]. Notably, more than 20% of CRC patients present distant metastases at the time of initial diagnosis, and fewer than 20% survive beyond five years of follow-up [17]. Therefore, more accurate predictive biomarkers are needed to guide treatment selection and improve OS in patients with recurrent and mCRC. Currently available biomarkers mainly include deficiency in mismatch repair (dMMR) and high microsatellite instability (MSI-H), *Human epidermal growth factor receptor 2* (HER2) amplification, and *RAS/BRAF* mutations. However, additional predictive biomarkers are urgently needed for proficient MMR (pMMR)/MSS CRC, which accounts for approximately 90% of cases, in order to optimize therapeutic decision-making [18,19]. These data underscore the need for more informative predictive biomarkers to refine treatment selection and improve outcomes in recurrent and mCRC.

Within the hierarchical framework proposed in this review, we explore the potential utility of metabolic biomarkers for identifying biologically distinct CRC subgroups that may benefit from tailored therapeutic strategies. CRC metabolic alterations do not represent equivalent pathways operating independently; rather, they can be organized into functionally distinct layers. Metabolic signaling kinases act as upstream nutrient- and energy-sensing hubs that connect oncogenic signaling with cellular metabolic demands. Core bioenergetic programs, including glycolysis and mitochondrial metabolism, provide an energetic and biosynthetic basis for tumor growth. Adaptive pathways, such as amino acid and lipid metabolism, support metabolic flexibility under environmental and therapeutic stress. Finally, autophagy and endocrine signals operate as context-dependent regulatory and systemic layers that further shape tumor adaptation.

We point out that the biomarkers discussed in this section are interpreted according to their position within this integrated immunometabolic network, rather than as isolated molecular alterations, and according to their functional relevance within CRC metabolic organization. First, we discussed metabolic signaling kinases not as primary metabolic drivers, but as upstream regulatory events that coordinate nutrient sensing, oncogenic cascades, and downstream metabolic rewiring. Then, we examine core bioenergetic programs, including glucose metabolism and mitochondrial metabolism, such as Tricarboxylic acid (TCA) cycle and oxidative phosphorylation (OXPHOS), followed by adaptive metabolic programs, such as amino acid and lipid metabolism. Finally, we discuss autophagy and metabolic hormones as context-dependent regulatory and systemic layers that modulate metabolic plasticity.

### 2.1. Metabolic Signaling Kinases

Dynamic metabolic adaptation is a hallmark of CRC tumorigenesis, supporting proliferative demands and contributing to therapeutic resistance. Central to this process is the dysregulation of metabolism-related protein kinases that integrate oncogenic signaling with metabolic control, providing both mechanistic insight and potential predictive biomarkers. Phosphoinositide 3-kinase (PI3K)/Protein kinase B (AKT)/Mechanistic target of rapamycin (mTOR) axis, AMP-activated protein kinase (AMPK), and pyruvate dehydrogenase kinases (PDKs) can be considered upstream regulators of CRC metabolic reprogramming. Indeed, they function as a central integrative network for nutrient and energy sensing. In particular, the PI3K/AKT/mTOR axis is one of the major pathways altered in CRC, and its dysregulation drives initiation, progression, metastasis, and drug resistance in this tumor type [20].

Moreover, oncogenic activation of PI3K and its downstream effector AKT promotes glycolysis, lipid biosynthesis, and anabolic growth, supporting CRC cell survival, proliferation, and invasion [21]. Analyses of CRC cell line panels revealed correlations between PI3K activity and drug-sensitivity profiles, evidencing that the PI3K/P21 (RAC1) activated kinase 1 (PAK1) axis may influence responses to PI3K inhibitors or other agents across molecularly heterogeneous CRC models. Indeed, PAK1-mediated downregulation of Mitogen-activated protein kinase (MAPK) signaling has been proposed as a potential biomarker for identifying patients who may benefit from the PI3K inhibitor copanlisib [22].

The overexpression of mTOR has been reported in CRC and correlates with poor prognosis and high tumor mutation burden (TMB). Increased mTOR activation has also been observed in microsatellite instability (MSI) CRC cell lines, suggesting a link between mTOR signaling and specific molecular subtypes [23]. These findings support mTOR activity as potential biomarker and therapeutic target. Clinical validation of PI3K/AKT/mTOR pathway alterations remains ongoing, with current trials evaluating combinations of pathway inhibitors with chemotherapy or immunotherapy to overcome resistance driven by metabolic adaptability.

AMPK is a central regulator of cellular energy homeostasis, activated by elevated AMP/ATP ratios and by shifting metabolism from anabolic to catabolic processes. Altered phosphorylation of AMPK subunits has been observed in CRC and may influence tumor progression. In particular, phosphorylation of the AMPKα1 subunit at serine 485 (S485), which inhibits AMPK activity, promotes proliferation, migration, and tumor growth in experimental models [24]. On the other hand, AMPK activation under glucose-restricted conditions, mediated by pyrophosphatase 1, enhances mitophagy through phosphorylation of Unc-51-like kinase 1 and FUN14 domain-containing protein 1, promoting OXPHOS and supporting tumor progression [25]. Clinically, increased AMPK expression correlates with lymph node involvement and distant metastasis, although its association with OS remains inconsistent [26]. These findings highlight the context-dependent role of AMPK as both a metabolic regulator and a modulator of tumor behavior.

PDKs, particularly PDK4, regulate the balance between glycolysis and mitochondrial metabolism by inhibiting the pyruvate dehydrogenase complex, thereby reducing oxidative glucose metabolism. This shift promotes glycolytic dependence, supporting proliferation, survival, and resistance to apoptosis across multiple cancer types [27]. In CRC, PDK4 enhances migration, invasion, and resistance to apoptosis by modulating the glycolytic-oxidative metabolic balance [28]. Its upregulation has been linked to increased resistance to chemotherapy and poor prognosis in metastatic CRC [29]. These findings suggest that PDK4 may serve as a predictive biomarker of treatment response.

Through this extensive metabolic crosstalk, these pathways contribute to the establishment of distinct tumor metabolic states associated with proliferation, metastatic dissemination, immune evasion, and therapy response. Consequently, their clinical relevance may extend beyond the prognostic value of individual biomarkers. Increasing evidence suggests that integrated metabolic kinase-based signatures, particularly those centered on PI3K pathway alterations and downstream metabolic rewiring, could improve patient stratification and prediction of treatment response in CRC [30]. The most relevant are reported in Table 1.

Taken together, these kinases establish the metabolic framework that coordinates and functionally integrates the downstream pathways involved in CRC progression, metastasis, and therapy resistance. Defining kinase activity in well-characterized patient cohorts and evaluating its predictive value in prospective studies will be essential for clinical translation. Furthermore, clarifying their context-dependent roles within the TME and under metabolic stress will further refine their utility as biomarkers and therapeutic targets in CRC.

### 2.2. Glucose Metabolism

Glucose metabolism represents one of the hallmarks of metabolic reprogramming in CRC, providing both the energy and biosynthetic intermediates required to sustain rapid tumor growth and progression. An increase in glucose uptake, aerobic glycolysis, and lactate production even under oxygen-replete conditions is commonly referred to as the Warburg effect [31,32]. Beyond ATP production, glycolytic intermediates fuel nucleotide, amino acid, and lipid biosynthesis while contributing to redox balance and TME remodeling. Consequently, dysregulated glucose metabolism constitutes one of the major contributors to CRC progression, therapy resistance, immune evasion, and metastatic dissemination [33]. Emerging evidence indicates that these alterations may serve as prognostic biomarkers and, in selected contexts, predictors of therapeutic response.

In this section, we discuss the role of the *Solute carrier family 2 member 1* (*SLC2A1*) gene, which encodes glucose transporter 1 (GLUT1), which mediates cellular glucose uptake and represents a key determinant of glycolytic metabolism in CRC. Although GLUT1 is not a signaling molecule itself, its expression is regulated by several oncogenic and metabolic pathways, including Kirsten rat sarcoma viral oncogene homolog (KRAS)/MAPK, PI3K/AKT/mTOR, and Hypoxia-inducible factor 1 alpha (HIF-1α) signaling. The expression of GLUT1 has been associated with advanced tumor stage, lymph node metastasis, and reduced OS in CRC [34], and is consistently higher in tumor tissues than in normal mucosa. Oncogenic *KRAS* mutations, present in approximately 50% of CRCs, may further enhance GLUT1 expression by activating the MAPK/extracellular signal-regulated kinase (ERK)/HIF-1α axis [35,36,37,38] and are independently linked to poorer prognosis in metastatic CRC [39]. However, it remains unclear whether GLUT1 expression provides independent prognostic value within *KRAS*-mutant tumors or primarily reflects downstream KRAS signaling. Consistent with this uncertainty, several meta-analyses have confirmed the association between GLUT1 overexpression and adverse clinical outcomes in CRC [34,35]. Preclinical evidence further indicates that Short transient receptor potential channel 5/GLUT1 overexpression contributes to chemoresistance by modulating calcium homeostasis and activating the Wnt/β-catenin signaling pathway [40]. In rectal cancer, elevated GLUT1 levels have also been linked to advanced stage, lymph node involvement, distant metastasis, and reduced responsiveness to radiotherapy and chemotherapy [41]. Despite these observations, the absence of prospective validation studies currently limits the clinical utility of GLUT1 as a predictive biomarker.

Concerning the enzymes involved in glycolysis, Hexokinase 2 (HK2) is a key regulator of glycolytic flux and mitochondrial metabolism, and its overexpression correlates with increased tumor aggressiveness, multidrug resistance, and epithelial–mesenchymal transition (EMT) in CRC [42]. Beyond its metabolic role, HK2 promotes tumor cell survival by interacting with mitochondrial voltage-dependent anion channels, thereby inhibiting apoptosis across multiple cancer types, including CRC [43]. Preclinical studies further implicate HK2 in chemotherapy resistance, particularly to oxaliplatin. Mechanistically, HK2 stabilizes the oncogenic transcription factor Twist1 by preventing its proteasomal degradation, thereby promoting Twist1-mediated resistance in CRC cells [42]. In addition, HK2-driven glycolytic activity may attenuate chemotherapy-induced oxidative stress and apoptosis. Also in this case, clinical validation remains limited.

Pyruvate kinase M2 (PKM2) serves dual functions in cellular metabolism and transcriptional regulation. In CRC, its upregulation correlates with advanced tumor stage, metastatic dissemination, and reduced OS [44]. PKM2 interacts with multiple signaling pathways to promote β-catenin activation and cell cycle progression; in particular, the β-catenin–PKM2 axis enhances the Warburg effect, driving metabolic rewiring and activation of oncogenic programs in CRC [45,46,47]. Elevated PKM2 expression further correlates with advanced TNM stage and adverse clinical outcomes, supporting its potential role as both a prognostic biomarker and a therapeutic target [48]. PKM2 has been implicated in chemotherapy resistance through mechanisms of metabolic adaptation [44]. Consistently, its inhibition enhances the efficacy of 5-fluorouracil (5-FU) in CRC cell lines and in vivo models [49]. Despite these findings, its prognostic and predictive value remains incompletely defined, and PKM2 is not currently used in routine clinical practice to guide treatment decisions.

Lactate dehydrogenase A (LDHA) represents another key glycolytic enzyme implicated in CRC progression. Its overexpression enhances lactate production and contributes to acidification of the TME, thereby promoting invasion and metastatic potential [50]. Experimental inhibition of LDHA markedly reduces cell proliferation in both in vitro and in vivo CRC models. Clinically, elevated total LDH levels and increased hypoxia-associated LDH isoenzymes correlate with adverse outcomes in CRC [51,52]. Serum LDH has therefore been proposed as a general prognostic marker in metastatic CRC, with high levels consistently associated with reduced OS [53,54]. In patients receiving anti-angiogenic therapies such as bevacizumab, elevated LDH levels have also been linked to shorter progression-free survival (PFS) and OS, likely reflecting hypoxia-driven angiogenic activity [55,56]. Despite these associations, LDH lacks tumor specificity and should be regarded as a nonspecific indicator of systemic metabolic and hypoxic stress rather than a CRC-specific biomarker.

Glycolytic reprogramming also shapes the response to immunotherapy in CRC. Elevated glycolytic activity, a hallmark of metabolic adaptation, is closely associated with immune evasion, resistance to ICIs, and unfavorable clinical outcomes. Increased glycolytic flux, often accompanied by lactate accumulation, contributes to the establishment of an immunosuppressive TME that impairs the activity of effector immune cells and limits the efficacy of immunotherapies [57,58,59]. GLUT1 overexpression should not be regarded as an isolated event, but rather as part of a broader metabolic network orchestrated by oncogenic signaling and adaptive stress responses in CRC. Increased glucose uptake and aerobic glycolysis functionally interact with PI3K/AKT/mTOR activation, hypoxia-associated pathways, amino acid metabolism, lipid biosynthesis, and mitochondrial rewiring to sustain rapid proliferation, metastatic dissemination, immune suppression, and therapeutic resistance [60]. In this regard, a study identified a five-gene glycolysis-related signature including *beta-enolase*, *glypican-1*, *prolyl 4-hydroxylase subunit alpha-1*, *sperm-associated antigen 4*, and *stanniocalcin-2*, which could improve prognostic stratification of CRC patients [61].

Glucose metabolism-related markers, including GLUT1, HK2, PKM2, and LDHA, consistently delineate aggressive CRC phenotypes and adverse clinical trajectories (Table 1). Despite strong biological plausibility and accumulating experimental and clinical evidence, their translation into routine clinical practice remains limited. This gap reflects tumor heterogeneity, lack of standardized assessment strategies, and insufficient prospective validation. Advancing the clinical utility of these biomarkers will require integrative approaches that combine metabolic profiling with genomic and immune features, thereby enabling more refined patient stratification and more effective therapeutic decision-making in CRC.

### 2.3. Amino Acid Metabolism

Beyond their role in protein synthesis, amino acids provide essential substrates for nucleotide and lipid biosynthesis, redox homeostasis, and epigenetic regulation. Dysregulated amino acid metabolism supports tumor proliferation and survival in CRC, particularly under conditions of metabolic stress and therapeutic pressure [62]. Amino acids such as tryptophan and glutamine provide nitrogen for nucleotide biosynthesis and can serve as alternative energy sources sustaining tumor growth [63]. Therefore, these pathways represent both therapeutic vulnerabilities and sources of metabolic biomarkers with potential prognostic and predictive relevance. Signatures based on amino acid metabolism-related genes have been associated with the immune TME and proposed as tools to predict prognosis and immunotherapy response in CRC [64], especially glutamine metabolism [65]. Additional Glutamine metabolism-related genes (GMRGs) have further refined risk prediction and provided insights relevant to clinical management [66]. In colon adenocarcinoma, a 10-GMRG prognostic subset developed by Yu et al. demonstrated independent predictive value for survival, TME characteristics, and immunotherapy responsiveness [67].

Similarly, the serine-glycine one-carbon (SGOC) metabolic network has acquired relevance [68]. Alterations in amino acid metabolism, particularly involving glutamine utilization and the SGOC pathway, reflect the metabolic state of CRC tumors and may serve as indicators of disease progression. In CRC, enzymes involved in serine biosynthesis, including phosphoglycerate dehydrogenase (PHGDH), phosphoserine aminotransferase 1 (PSAT1), and phosphoserine phosphatase (PSPH), are frequently upregulated and correlate with aggressive clinicopathological features. PHGDH-driven metabolic flux enhances nucleotide synthesis, anabolic growth, and redox buffering, promoting tumor cell survival and resistance to 5-FU [69]. PSAT1 upregulation supports proliferation and invasion and correlates with advanced disease stage, identifying it as a potential marker of poor prognosis [70], while PSPH has been implicated in CRC progression, where increased expression supports tumor growth through autophagy-dependent mechanisms and may influence antitumor immunity [71,72].

Among the pathways that establish a direct link between amino acid metabolism and therapeutic response, particularly in the context of immunotherapy, there is tryptophan catabolism through kynurenine. Indoleamine 2,3-dioxygenases (IDO1/2) and tryptophan 2,3-dioxygenase 2 (TDO2) catalyze the rate-limiting steps of this pathway, generating kynurenine and downstream immunoregulatory metabolites [73]. Elevated IDO expression correlates with immune tolerance, metastatic progression, and poor prognosis, while an increased serum kynurenine/tryptophan ratio has been proposed as a screening marker [74]. Gene-based models derived from tryptophan metabolism pathways have been validated across independent CRC cohorts, supporting their potential as predictive metabolic biomarkers [75]. However, their clinical applicability in MSS CRC requires rigorous prospective validation.

Tumor metabolic flexibility is further supported by upregulation of amino acid transporters, which increases substrate availability for protein synthesis, nucleotide biosynthesis, redox balance, and activation of oncogenic pathways, such as mTOR [76]. Among these, L-type amino acid transporter 1 (LAT1; *SLC7A5* gene) has emerged as a clinically relevant candidate. LAT1 functions as a sodium-independent antiporter of large neutral amino acids. It forms a heterodimer with 4F2hc (*SLC3A2* gene), enabling plasma membrane localization and efficient amino acid uptake. High expression of LAT1 correlates with tumor aggressiveness, metastatic potential, and reduced OS in patients undergoing surgical resection [77]. Stage-specific analyses further suggest a stronger association in early-stage disease, indicating that amino acid transport pathways may have translational relevance [78].

We can summarize that amino acid metabolism supports CRC adaptation to energetic stress and therapy-induced damage by coordinating the regulation of metabolic enzymes and transport systems (Table 1). Alterations in these pathways correlate with tumor aggressiveness, survival outcomes, and therapeutic response, supporting their potential role as prognostic biomarkers. Their integration into clinical practice will require validation in large prospective cohorts and the development of standardized assessment strategies.

### 2.4. Lipid Metabolism

CRC exhibits marked metabolic adaptability, with lipid metabolism emerging as one of the most consistently rewired programs. Beyond structural roles, lipids act as signaling mediators and energy sources (e.g. oxidative stress tolerance and susceptibility to ferroptosis), shaping the TME and influencing immune function and therapy response [79,80].

De novo lipogenesis represents a central axis of lipid metabolic configuration. Fatty acid synthase (FASN) catalyzes the synthesis of palmitate from acetyl-CoA and malonyl-CoA, providing substrates for membrane biosynthesis, protein acylation, and lipid-mediated signaling. High levels of FASN correlate with aggressive tumor phenotypes and adverse clinical outcomes. Increased phosphatidylcholine production driven by FASN contributes to membrane remodeling and tumor progression [81]. FASN-dependent lipid synthesis also influences therapeutic response. Enhanced lipogenesis has been linked to oxaliplatin resistance, whereas combined treatment with FASN inhibitors and oxaliplatin increases cell cycle arrest and apoptosis through inhibition of MAPK/ERK and PI3K/AKT signaling pathways [82]. FASN further regulates cancer stemness and ferroptosis resistance through Sterol regulatory element-binding protein 2 (SREBP2)-dependent mechanisms [83]. In addition, genetic polymorphisms in FASN have been associated with clinical outcomes in patients receiving bevacizumab-based therapy, supporting a link between lipid metabolism and resistance to anti-VEGF treatment [84]. These observations position FASN as a biomarker associated with aggressive disease behavior, reduced ferroptotic susceptibility, and altered therapeutic responsiveness.

Analogously, Acyl-CoA synthetase long-chain family member 4 (ACSL4) links lipid remodeling to ferroptosis sensitivity. This enzyme regulates the incorporation of arachidonic and adrenic acids into membrane phospholipids, generating substrates prone to lipid peroxidation. ACSL4 is mechanistically relevant because it links lipid remodeling to ferroptotic vulnerability, inflammatory signaling, immune regulation, and therapy response. By enriching cellular membranes with polyunsaturated phospholipids, ACSL4 increases the availability of lipid substrates prone to iron-dependent peroxidation, thereby acting as a major determinant of ferroptosis sensitivity [85]. ACSL4-dependent lipid peroxidation can modulate oxaliplatin sensitivity in CRC. Indeed, the abnormal activation of cyclin-dependent kinase 1 induces ACSL4 phosphorylation and promotes its polyubiquitination and degradation via Ubiquitin protein ligase E3 component N-recognin 5. Down-regulation of ACSL4 decreases polyunsaturated fatty acid (FA) biosynthesis, inhibits lipid peroxidation and ferroptosis, and increases oxaliplatin resistance [86]. On the other hand, an increase in the levels of ACSL4 correlates with unfavorable clinical outcomes. Experimental depletion of ACSL4 reduces tumor growth and alters immune cell infiltration in immunocompetent models, highlighting the interplay between lipid metabolism and antitumor immunity [87]. Therefore, ACSL4-dependent lipid remodeling influences both ferroptotic vulnerability and immune-mediated tumor control, supporting its potential as a biomarker of response to therapies that rely on oxidative stress or immune activation [88].

Other phospholipid remodeling enzymes such as lysophosphatidylcholine acyltransferase 1 (LPCAT1) further regulate ferroptosis susceptibility by shaping membrane lipid composition. LPCAT1 increases phospholipid saturation, promoting resistance to lipid peroxidation and enabling tumor cells to evade ferroptotic cell death in CRC and other malignancies [89]. This function has direct therapeutic implications, as LPCAT1 expression may influence sensitivity to ferroptosis-inducing strategies currently explored to overcome chemoresistance. LPCAT1, either considered a single marker or within lipid peroxidation gene signatures, may help identify tumors with reduced susceptibility to cell death. Despite strong biological plausibility and growing translational evidence, ACSL4 and LPCAT1 are not yet clinically validated.

Lipid uptake and transport pathways provide an additional layer of metabolic flexibility in CRC. Tumor, stromal, and immune cells within the TME can acquire exogenous FAs and cholesterol through specific transporters and scavenger receptors, allowing tumors to bypass de novo synthesis under metabolic stress or therapeutic pressure [90]. Cluster of differentiation 36 (CD36) is among the most extensively studied lipid uptake receptors and has been linked to metastatic dissemination and therapy resistance in several tumor types, including CRC [91]. CD36-mediated lipid acquisition influences both tumor progression and therapeutic sensitivity, although CRC-specific clinical evidence remains heterogeneous [92,93].

FA oxidation (FAO)-related enzymes are increasingly recognized as important mediators of metabolic plasticity. In CRC, they contribute to energy production, redox homeostasis, metastatic adaptation, and therapy resistance. Mechanistically, FAO provides ATP through mitochondrial β-oxidation of FA, thereby supporting tumor cell survival under nutrient deprivation, oxidative stress, and therapeutic pressure. Among the principal FAO-related enzymes, carnitine palmitoyltransferase 1A (CPT1A), the rate-limiting enzyme controlling mitochondrial FA transport, has emerged as a major regulator of tumor progression and metabolic adaptation. Increased CPT1A expression promotes CRC cell proliferation, stemness, EMT, and metastatic dissemination by inhibiting anoikis [94]. Moreover, FAO activity functionally interacts with PI3K/AKT/mTOR signaling and lipid remodeling pathways, affecting survival and therapeutic stress [95]. The activation of FAO-related pathways within immune cell subsets of the CRC microenvironment has been linked to immunosuppressive phenotypes. Increased FAO activity in tumor-associated plasmacytoid dendritic cells (DCs) promotes immune suppression, whereas pharmacological FAO inhibition restores antitumor activity and delays tumor progression [96,97]. These findings indicate that FAO-related gene expression reflects both tumor-intrinsic metabolic states and immune metabolic adaptation, influencing responsiveness to immunotherapy. Transcriptomic models based on mitochondrial lipid metabolism further support this concept, linking FAO signatures to immune infiltration patterns and immunosuppressive TME features in high-risk CRC patients [98].

Other transcriptomic studies have demonstrated that gene signatures associated with FA metabolism can be useful to stratify patients affected by CRC or colorectal adenocarcinoma (COAD) [99,100,101], further confirming that integrated lipid metabolic signatures could better predict prognosis and therapy response than individual biomarkers.

Lipid metabolism is transitioning from a descriptive hallmark of CRC biology to a source of clinically relevant biomarkers. Alterations in de novo lipogenesis, ferroptosis-associated lipid remodeling, and FAO-related pathways delineate distinct metabolic states linked to tumor aggressiveness and therapeutic resistance (Table 1). Defining these states within well-annotated patient cohorts and integrating lipid metabolic markers with established molecular classifiers, including MSI status and *RAS/BRAF* alterations, will determine their clinical utility in refining patient stratification and guiding treatment selection.

### 2.5. TCA Cycle and OXPHOS Metabolism

Beyond glycolysis, mitochondrial metabolism and the TCA cycle remain functionally active and critically support tumor growth, redox balance, and biosynthetic processes [102,103]. Oxidative metabolism is frequently reprogrammed in CRC to sustain anabolic demands and confer metabolic flexibility promoting cell spread and metastasis [104]. Increasing evidence suggests that alterations in TCA cycle enzymes, including citrate synthase (CS), isocitrate dehydrogenase (IDH), succinate dehydrogenase (SDH), and fumarate hydratase (FH), are consistently associated with clinical outcomes and represent prognostic candidates.

CS catalyzes the condensation of acetyl-CoA and oxaloacetate to form citrate, initiating the TCA cycle. CS overexpression correlates with tumor progression and metastatic potential across multiple cancer types [105]. In CRC, alterations in CS expression and splicing emerged as clinically relevant features. A CRC-specific splice variant, CS-ΔEx4, is markedly upregulated in tumor tissues compared with full-length CS, and its expression correlates with increased recurrence rates and poorer survival outcomes. This isoform promotes metabolic rewiring, leading to accumulation of oncometabolites and epigenetic alterations that support oncogenic gene expression [102]. Functional studies further indicate that CS activity influences proliferation, apoptosis, mitochondrial function, and ATP production. CS knockdown reduces proliferation, increases apoptosis, and impairs mitochondrial membrane potential in several cancer cells, including CRC [106]. In addition, post-translational regulation of CS through SIRT5-mediated desuccinylation enhances colon cancer cell proliferation and migration, indicating that CS activity is regulated at multiple levels, such as alternative splicing and post-translational modifications [105]. These findings suggest CS, and particularly the CRC-specific CS-ΔEx4 isoform, as a promising biomarker linking TCA cycle alterations to clinical outcomes and therapeutic susceptibility.

Considering alterations of other TCA cycle enzymes, although IDH mutations are uncommon in CRC compared with gliomas or cholangiocarcinomas, dysregulation of IDH expression and α-KG metabolism has been reported [107]. Upregulation of IDH2 in CRC tissues and cell lines correlates with enhanced tumor growth and adverse clinical outcomes, supporting its role as a tumor-promoting metabolic factor [108,109]. Post-translational modification of IDH1 also appears to influence tumor behavior. For example, hyperacetylation of IDH1 at lysine 224 (K224) correlates with distant metastasis and reduced survival in patients, whereas IDH1 deacetylation suppresses invasion and migration in experimental models [110]. In addition, exosomal IDH1 has been shown to enhance resistance to 5-FU, suggesting a role in mediating chemotherapy resistance [111]. These features converge in promoting tumor aggressiveness and reducing sensitivity to chemotherapy.

Focusing on SDH and FH, SDH is unique among TCA cycle enzymes because it participates in both the TCA cycle and the electron transport chain as complex II, while FH exerts important tumor-suppressive functions. Dysregulation of either enzyme promotes accumulation of oncometabolites that favor cancer progression through HIF signaling, epigenetic remodeling, and broader metabolic rewiring [112,113]. Specifically, SDH impairment leads to succinate accumulation, a recognized oncometabolite that sustains pro-tumorigenic signaling and aggressive tumor behavior [114]. Deficiency of the SDH subunit C has been linked to worse prognosis in CRC, metastatic dissemination, and metabolic reprogramming that supports tumor progression [115]. On the other hand, reduced FH activity has likewise been associated with adverse prognosis [116]. Loss of FH function and fumarate accumulation promote EMT and invasive phenotypes through epigenetic mechanisms, including inhibition of α-KG-dependent dioxygenases [117]. Conversely, increased FH expression enhances the efficacy of anti-programmed cell death protein 1 (PD-1) treatment in CRC models by improving antitumor immune responses [116,117,118]. These observations support the idea that downregulation of SDH and FH marks biologically aggressive CRC subsets and may influence both prognosis and therapeutic response.

Transcriptomic analyses have identified CRC subsets characterized by elevated mitochondrial respiration and increased expression of TCA cycle-related genes. High OXPHOS activity correlates with enhanced metastatic potential, resistance to chemotherapy, and reduced OS [119]. CRC tumors with increased mitochondrial metabolic activity also display resistance to 5-FU in experimental models, linking OXPHOS dependence to therapeutic vulnerability [120].

Increased expression of Peroxisome proliferator-activated receptor gamma coactivator 1 alpha (PGC-1α) is associated with enhanced mitochondrial biogenesis and OXPHOS activity in CRC, promoting metastatic potential and unfavorable clinical outcomes. PGC-1α functions as a transcriptional coactivator that interacts with multiple transcription factors to coordinate metabolic remodeling [121]. Its upregulation supports an OXPHOS-dependent survival program, which is further enhanced by Sirtuin 1 (SIRT1)-mediated deacetylation of PGC-1α forming an axis that overcomes chemotherapy resistance [122].

An elevated expression of NADH dehydrogenase 1 alpha subcomplex subunit 4-like 2 (NDUFA4L2) has been associated with a decrease in OS, disease progression, metastatic dissemination, and resistance to 5-FU [123,124], and high levels of this enzyme correlate with immune cell infiltration patterns, and receiver operating characteristic analyses support its potential as a candidate biomarker [125].

Although aerobic glycolysis is a dominant feature of CRC metabolism, increasing evidence indicates that OXPHOS-dependent phenotypes may identify clinically relevant tumor states characterized by cancer stemness, metastatic potential, immune modulation, and resistance to chemotherapy or targeted therapy. This suggests that TCA/OXPHOS-related markers may have greater prognostic and predictive value when evaluated as integrated mitochondrial or energy-metabolism signatures. Interestingly, many studies have developed OXPHOS- and TCA cycle/carbon-related signatures in CRC, COAD, and other malignancies that stratify patients according to survival, metastatic potential, tumor immune microenvironment features, and therapeutic vulnerabilities [126,127,128,129].

Alterations in TCA cycle enzymes and mitochondrial metabolism define an important axis of metabolic plasticity. Dysregulation of CS, IDH1/2, SDH, and FH, together with activation of OXPHOS-related programs, is consistently linked to tumor progression and adverse clinical outcomes (Table 1). These findings support the use of mitochondrial metabolic signatures as complementary biomarkers of CRC aggressiveness, metabolic adaptation, and treatment response.

### 2.6. Autophagy

Autophagy is a conserved lysosomal degradation pathway that maintains cellular homeostasis by recycling proteins and organelles under conditions of hypoxia, nutrient deprivation, and chemotherapy exposure. In CRC, autophagy exerts a context-dependent role: it may suppress malignant transformation during early tumorigenesis, while supporting tumor cell survival, invasion, and therapeutic resistance in advanced disease. These features provide a strong rationale for investigating autophagy-related pathways as biomarkers of prognosis and drug response, as well as potential therapeutic targets [130].

Canonical autophagy markers, including microtubule-associated protein 1 light chain 3 B (MAP1LC3B), Beclin-1 (BECN1), and Sequestosome-1 (p62/SQSTM1) have been extensively studied in CRC. BECN1 overexpression correlates with reduced survival in patients receiving chemotherapy, while MAP1LC3B upregulation has been linked to decreased OS, particularly in *KRAS*-mutant CRC [131,132]. Conversely, reduced expression of BECN1 and MAP1LC3B has also been associated with aggressive tumor phenotypes and metastatic progression [133]. These seemingly discordant findings likely reflect differences in tumor stage, molecular context, and methodological variability across studies [134,135]. Evaluating combined autophagy markers rather than individual proteins may therefore improve predictive accuracy and generate more robust prognostic models.

Additional autophagy-related genes, including Autophagy-related gene 2B (ATG2B), ATG4B, and ATG16L2, have been investigated as potential biomarkers. ATG2B, a lipid transfer protein required for autophagosome formation, has been linked to clinical outcomes in CRC, with the rs17094017 polymorphism associated with improved OS in patients undergoing chemotherapy [136]. ATG4B, a cysteine protease involved in MAP1LC3B processing and autophagosome maturation, promotes autophagic flux and tumor cell survival under stress conditions, contributing to chemoresistance. Elevated ATG4B expression correlates with tumor aggressiveness and unfavorable survival outcomes, supporting its relevance as a potential therapeutic target [137]. ATG16L2, a component of the autophagy elongation complex, has been associated with CRC prognosis and its increase correlates with more favorable clinical outcomes, suggesting that specific ATGs may distinguish biologically distinct CRC subgroups with divergent prognostic trajectories [138].

Multi-gene signatures based on autophagy-related genes have been developed using datasets from The Cancer Genome Atlas (TCGA), Human Autophagy-dedicated Database, and Gene Expression Omnibus (GEO) and validated across independent cohorts. These transcriptomic datasets show strong associations with OS, disease-free survival, early relapse, tumor immune infiltration, and chemotherapy sensitivity [139,140,141,142,143,144,145,146]. Such findings indicate that ATG signatures may outperform single-marker approaches, particularly when integrated with clinical staging systems and molecular classifiers.

Because autophagy requires lysosomal fusion for cargo degradation, lysosome-associated membrane proteins (LAMPs) provide additional insight beyond upstream autophagy markers. Expression of LAMP1 and LAMP2, in combination with MAP1LC3B and BECN1, correlates with tumor invasiveness, tumor budding, and patient survival, supporting their role as composite biomarkers [147]. The chaperone-mediated autophagy regulator LAMP2A has been implicated in chemoresistance, and its elevated expression promotes tumor progression and resistance to cisplatin through autophagy-dependent mechanisms [148].

Autophagy-related biomarkers, particularly multi-gene and pathway-based models, provide a framework for improved patient stratification and prediction of therapeutic response in CRC (Table 1). Their clinical implementation will depend on prospective validation and methodological standardization. Integration of autophagy-related signatures with molecular and clinical parameters may enhance risk assessment and support more personalized therapeutic strategies in CRC.

### 2.7. Metabolic Hormones in CRC

Although metabolic hormone signaling is generally considered secondary to the core cell-intrinsic metabolic pathways previously described, hormones such as insulin, Insulin-like growth factor (IGF-1), leptin, adiponectin, and glucagon-like peptides play an important modulatory role in CRC by influencing systemic energy balance, nutrient availability, inflammation, and TME interactions.

CRC progression is closely intertwined with systemic metabolic regulation. In addition to cell-intrinsic metabolic rewiring, tumor cells respond to circulating endocrine signals that govern nutrient utilization, inflammation, and energy homeostasis. Metabolic hormones including pancreatic hormones, components of the IGF axis, adipose-derived factors and glucagon-related signaling converge on pathways that regulate glucose metabolism, mitochondrial function, lipogenesis, oxidative stress responses, and immune stromal interactions. Because many of these factors are readily measurable in circulation, metabolic hormones represent attractive candidates for biomarker development in CRC.

Hyperinsulinemia and IGF signaling promote proliferative and anti-apoptotic programs through receptor tyrosine kinases and downstream PI3K/AKT and MAPK pathways, thereby supporting anabolic metabolism and tumor cell survival under stress conditions. Elevated circulating IGF-1 levels have been associated with increased risk, supporting a potential causal role of the IGF axis in colorectal carcinogenesis [149]. However, other studies have reported no significant association, while increased plasma levels of Insulin-like growth factor binding protein (IGFBP) 2 correlate with higher tumor grade [150]. Clinical investigations have further explored the prognostic relevance of IGF-related factors. In mCRC, plasma levels of IGF-1, IGFBP3, IGFBP7, C-peptide, and adiponectin have been analyzed in relation to OS and PFS. While IGF-1, C-peptide, and adiponectin showed no significant association with OS, higher IGFBP3 and lower IGFBP7 levels were associated with improved survival [151]. In addition, tumor tissue analyses revealed IGF-1 expression in CRC compared with normal mucosa, while IGFBP3 upregulation correlated with lymph node metastasis and reduced five-year survival [152]. Also, the components of the IGF axis may hold clinical relevance, particularly in advanced disease.

Leptin and adiponectin are key adipose-derived hormones with partially opposing metabolic and inflammatory functions. In CRC, these adipokines modulate tumor biology through receptors expressed in tumor and stromal compartments. Increased leptin expression and reduced adiponectin receptor levels have been associated with metastatic progression [153]. Experimental studies further demonstrate that these hormones directly influence gene expression programs and malignant phenotypes in colon cancer cells, linking obesity-associated endocrine signaling to tumorigenesis [154]. Circulating adipokines show potential clinical relevance. Elevated serum leptin and non-high-molecular-weight (non-HMW) adiponectin have been associated with increased CRC risk, whereas high levels of HMW adiponectin have been observed following chemotherapy [155]. Additional studies suggest that markers of nutritional status and adipokines, including visfatin and resistin, correlate with chemotherapy response in advanced CRC. Higher albumin and pre-albumin levels are associated with improved outcomes, whereas elevated visfatin and resistin levels correlate with reduced treatment efficacy [156]. Visfatin has also been shown to promote chemoresistance through upregulation of multidrug resistance protein 1, linking metabolic signaling to drug resistance mechanisms [157].

Glucagon-related signaling is emerging as a potential endocrine–metabolic axis in CRC, linking systemic metabolic alterations, T2DM-associated hyperglucagonemia, nutrient availability, and intracellular signaling pathways. Experimental evidence indicates that glucagon promotes colon cancer cell proliferation, whereas glucagon receptor (GCGR) silencing attenuates cell growth. Mechanistically, these effects appear to involve GCGR-dependent modulation of AMPK and MAPK signaling, supporting a role for glucagon in CRC progression under metabolically altered conditions [158]. Given the central role of AMPK and MAPK in coordinating cellular energetics, stress adaptation, and proliferative responses, glucagon signaling may influence multiple biological processes relevant to tumor development. Other members of the glucagon family, particularly glucagon-like peptide-1 (GLP-1), have also been associated with immune infiltration, MSI, TMB, and immunotherapy-related features in pan-cancer and CRC-focused analyses, suggesting that endocrine signaling may contribute to both tumor behavior and immune contexture [159].

Although findings regarding individual endocrine biomarkers are sometimes inconsistent across studies, a common theme emerges in which endocrine–metabolic signaling influences CRC progression primarily through modulation of nutrient sensing, inflammation, immune regulation, and metabolic adaptation rather than through isolated hormone-specific effects. Within the hierarchical framework proposed in this review, metabolic hormones should be considered systemic modulators of CRC biology rather than primary determinants of cellular metabolic activity. Hormone-dependent pathways, including insulin/IGF signaling, leptin, adiponectin, glucagon/GLP-1 signaling, and other adipokine networks, integrate obesity, T2DM, nutrient availability, inflammation, and tumor–microenvironment interactions with disease progression (Table 1). Although these pathways operate downstream of the core bioenergetic programs that sustain tumor growth, they substantially influence cellular signaling, mitochondrial activity, immune regulation, metastatic potential, and treatment response. Consequently, their clinical value may be greater when assessed as components of integrated endocrine–metabolic signatures rather than as isolated circulating or tissue biomarkers. Emerging evidence supports the prognostic relevance of insulin/IGF-axis components, adipokines, and glucagon-related genes in CRC; however, clinically validated hormone-based signatures remain limited and require further prospective evaluation.

**Table 1:** Putative prognostic and drug response metabolic biomarkers for CRC.

Gene Acronym	Protein	Functional Category	Expression Activation	Prognosis	Drug Response	Functional State	References
*PIK3CA*; *PAK1*	Phosphatidylinositol-4,5-bisphosphate 3-kinase catalytic subunit α; P21 (RAC1) activated kinase 1	PI3K/PAK1 axis	High	Poor	Patient selection for copanlisib treatment	Oncogenic nutrient-sensing/anabolic state	[22]
*MTOR*	Mechanistic target of rapamycin	mTOR pathway	High	Poor	NA	Anabolic growth and immune-modulatory state	[23]
*PRKAA1* and related genes	Protein kinase AMP-activated catalytic subunit alpha 1 (AMPK)	Energy sensor	Low/high	Good/Poor (debated)	NA	Energy-stress adaptive state	[24,25,26]
*PDK4*	Pyruvate dehydrogenase kinase 4	Glucose and fatty acid metabolism	High	Poor	Resistance to chemotherapy	Glycolytic–oxidative switch/resistant state	[28,29]
*SLC2A1*	Solute carrier family 2 member 1	Glucose transporter (GLUT1)	High	Poor	Resistance to chemotherapy	Glycolytic proliferative state	[34,35,40,41]
*HK2*	Hexokinase 2	Glycolysis	High	Poor	Multidrug resistance	Glycolytic survival/resistant state	[42]
*PKM*	Pyruvate kinase M1/2	Glycolysis	High	Poor	Resistance to chemotherapy	Glycolytic proliferative and EMT-associated state	[44,48,49]
*LDH*	Lactate dehydrogenase	Glycolysis	High	Poor	Resistance to anti-angiogenic agents	Lactate-driven immunosuppressive state	[51,52,53,54,55,56]
Glycolysis-related genes (*ENO3*, *GPC1, P4HA1*, *SPAG4*, and *STC2*)	Enolase 3, Glypican-1, Prolyl 4-hydroxylase subunit alpha-1, Sperm-associated antigen 4, Stanniocalcin-2	Glycolysis	High	Poor	NA	Glycolytic aggressive/prognostic state	[61]
Amino acid metabolism-related genes		Amino acid metabolism	Low	Poor	Resistance to immunotherapy	Amino acid-depleted immunosuppressive state	[64]
Glutamine metabolism-related genes	ND	Amino acid metabolism	Differentially expressed	Poor/good	Resistance to chemo- and immuno-therapy	Glutamine-dependent adaptive/resistant state	[65,66,67]
*PHGDH*	Phosphoglycerate dehydrogenase	Serine biosynthesis	High	Poor	Resistance to chemotherapy	Serine/one-carbon stress-resistant state	[69]
*PSAT1*	Phosphoserine aminotransferase 1	Serine biosynthesis	High	Poor	NA	Serine biosynthesis proliferative/invasive state	[70]
*PSPH*	Phosphoserine Phosphatase	Serine biosynthesis	High	Poor	Antitumor immunity modulator	Serine-autophagy immune-modulatory state	[71,72]
*IDO1/2*	Indoleamine 2,3-dioxygenase 1/2	Tryptophan catabolism	High	Poor	NA	Tryptophan-kynurenine immunosuppressive state	[74]
Tryptophan metabolism-related genes	ND	Tryptophan catabolism	High	Poor	Antitumor immunity modulator	Tryptophan-driven immune-tolerant state	[75]
*SLC7A5*	Solute carrier family 7 member 5	Amino acid transporter	High	Poor	NA	Amino acid uptake/anabolic state	[77,78]
*FASN*	Fatty acid synthase	lipogenesis	High	Poor	Resistance to chemotherapy and anti-angiogenic agents	Lipogenic proliferative/resistant state	[81,82,84]
*ACSL4*	Acyl-CoA synthetase long chain family member 4	Lipid metabolism	High	Poor	Immune cytotoxicity modulator	Ferroptosis-linked lipid-remodeling state	[87]
*LPCAT1*	Lysophosphatidylcholine Acyltransferase 1	Membrane remodeling	High	Poor	NA	Ferroptosis-resistant membrane-remodeling state	[89]
*CD36*	Cluster of differentiation 36	lipid uptake receptor	High	Poor	Resistance to chemotherapy	Lipid uptake metastatic/resistant state	[92,93]
*CPT1A* and FAO-related genes	Carnitine palmitoyltransferase-1A	Lipid metabolism	High	Poor	Resistance to immunotherapy	FAO-dependent immunosuppressive state	[96,97,98]
Lipid metabolism-related genes	ND	Lipid metabolism	Differentially expressed	Poor	Resistance to immunotherapy	Lipid-adaptive immune-resistant state	[99,100,101]
*CS*	Citrate synthase	Krebs cycle	High	Poor	Therapy resistance	TCA-rewired aggressive state	[102,105]
*IDH1/2*	Isocitrate dehydrogenase 1/2	Krebs cycle	High	Poor	Resistance to chemotherapy	Oncometabolic chemotherapy-resistant state	[108,109,111]
*SDH*	Succinate dehydrogenase	Krebs cycle	Low	Poor	NA	Succinate-driven invasive/metastatic state	[115]
*FH*	Fumarate hydratase	Krebs cycle	Low	Poor	Modulation of ICI treatment	Fumarate/immune-response modulatory state	[116,117]
*PPARGC1A*	Peroxisome proliferator-activated receptor gamma coactivator 1α	Mitochondrial metabolic remodeling	High	Poor	Resistance to chemotherapy	OXPHOS-dependent metastatic/resistant state	[121,122]
*NDUFA4L2*	NADH dehydrogenase 1 alpha subcomplex subunit 4-like 2	OXPHOS	High	Poor	Resistance to chemotherapy	Hypoxia-associated OXPHOS-resistant state	[123,124,125]
OXPHOS-related genes	ND	OXPHOS	Differentially expressed	Poor	Resistance to chemotherapy and better response to immunotherapy	Mitochondrial oxidative/resistant state	[126,127,128,129]
*BECN1*	Beclin 1	Autophagy	High	Poor	Resistance to chemotherapy	Autophagy-dependent survival/resistant state	[131]
*MAP1LC3B*	Microtubule-associated protein 1 light chain 3 beta	Autophagy	High	Poor	NA	Autophagy-associated aggressive state	[132]
*BECN1*; *MAP1LC3B*	Beclin 1; Microtubule-associated protein 1 light chain 3 beta	Autophagy	Low	Poor	NA	Autophagy-defective aggressive state	[133]
*ATG2B*	Autophagy related 2B	Autophagy	ATG2B rs17094017 polymorphism	Good	Increased chemotherapy efficacy	Autophagy-associated chemosensitive state	[136]
*ATG4B*	Autophagy related 4B Cysteine Peptidase	Autophagy	High	Poor	Resistance to chemotherapy	Autophagy-driven chemoresistant state	[137]
*ATG16L2*	Autophagy related 16 like 2	Autophagy	High	Good	NA	Autophagy-associated favorable state	[138]
Autophagy-related genes	ND	Autophagy	Differentially expressed	Poor/good	Chemo- and immuno-therapy predictors	Autophagy-dependent prognostic/resistant state	[139,140,141,142,143,144,145,146]
*LAMP1/2*; *BECN1*; *MAP1LC3B*	Lysosomal associated membrane protein 1/2; Beclin 1; Microtubule-associated protein 1 light chain 3 beta	Autophagy-related pathway	High	Poor	NA	Lysosomal-autophagy invasive state	[147]
*LAMP2A*	Lysosome-Associated membrane protein 2A	Autophagy-related pathway	High	Poor	Resistance to chemotherapy	Chaperone-mediated autophagy resistant state	[148]
*IGF-1*	Insulin-like growth factor 1	metabolic factor	High (plasma)	CRC risk (debated)	NA	Systemic insulin/IGF anabolic state	[149]
*IGFBP2*	Insulin growth factor binding protein 2	metabolic factor	High (plasma)	Tumor grade	NA	Endocrine metabolic aggressive state	[150]
*IGFBP3*	Insulin growth factor binding protein 3	metabolic factor	High (plasma); high mRNA levels	Good (plasma); Poor (mRNA)	NA	Context-dependent IGF-modulatory state	[151,152]
*IGFBP7*	Insulin growth factor binding protein 7	metabolic factor	Low (plasma)	Good	NA	IGF-axis protective/modulatory state	[151]
*LEPR*	Leptin receptor	Metabolic homeostasis	High	Poor	NA	Leptin-driven inflammatory/pro-tumor state	[153]
*ADIPOR*	Adiponectin receptor	Metabolic homeostasis	Low	Poor	NA	Adiponectin-deficient inflammatory state	[153]
*LEP*; *ADIPOQ*	Leptin and adiponectin	Metabolic homeostasis	High (serum)	Poor	NA	Adipokine-imbalanced systemic state	[155]
*PBEF1*; *RETN*	Pre-B-cell colony-enhancing factor 1/visfatin and resistin	Metabolic homeostasis	High (serum)	Poor	Resistance to chemotherapy	Adipokine-driven chemoresistant inflammatory state	[156,157]
GLP-1-related genes	ND	metabolic homeostasis	Low	Poor	NA	Endocrine metabolic immune-modulatory state	[159]

ND: not defined; NA: not available.

Taken together, the findings discussed in this section highlight the importance of metabolic alterations in CRC while underscoring the gap that still separates biological evidence from clinical implementation. At present, routine therapeutic decision-making remains largely guided by established molecular biomarkers, including *RAS* and *BRAF* mutations, MSI/MMR status, *HER2* amplification, and selected gene fusions, which direct the use of anti-EGFR therapies, ICIs, BRAF-targeted strategies, and HER2-directed treatments [160,161]. By contrast, metabolism-associated biomarkers, including glycolytic enzymes, OXPHOS regulators, amino acid- and lipid-metabolism pathways, nutrient-sensing kinases, and autophagy-related proteins, remain largely investigational. Their most immediate clinical utility may lie in prognostic stratification, particularly when incorporated into multidimensional molecular signatures. Indeed, several transcriptomic studies have generated risk models associated with survival, immune-cell composition, metastatic behavior, and therapeutic responsiveness. Nevertheless, most of these signatures have been derived from retrospective TCGA- and GEO-based datasets and still require methodological standardization, independent multicenter validation, and prospective clinical assessment before they can be translated into routine clinical practice.

Summarizing the clinical perspectives, among the biomarkers discussed, only a limited number currently show evidence approaching clinical applicability. Serum LDH, metabolic gene signatures, OXPHOS-related signatures, amino acid metabolism-related signatures and selected immunometabolic transcriptomic models have demonstrated reproducible associations with prognosis or therapeutic response across independent cohorts. By contrast, many individual markers, including HK2, PKM2, ACSL4, LPCAT1, ATG4B, and several endocrine–metabolic factors, remain exploratory and require prospective validation before clinical implementation.

### 2.8. Convergence of Metabolic Programs into Functional Immunometabolic Configurations

**Figure 2:**
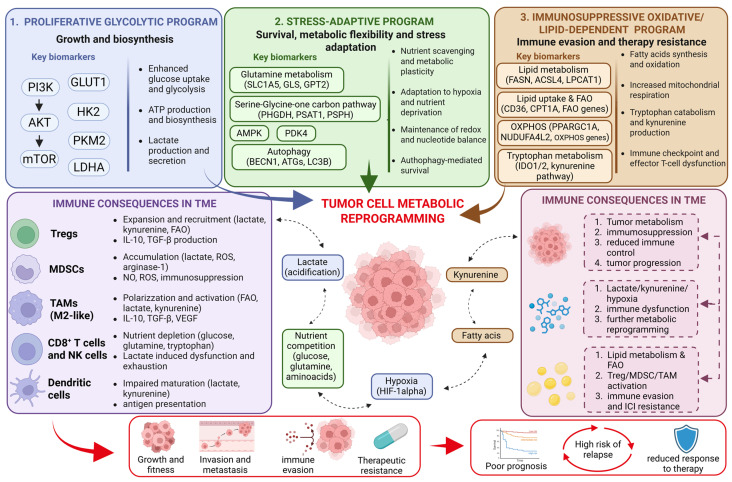
Convergence of metabolic programs into functional immunometabolic configurations in CRC. Glycolysis, amino acid metabolism, lipid metabolism, mitochondrial function, autophagy, and endocrine–metabolic signaling interact to generate distinct immunometabolic states associated with tumor progression, immune suppression, and therapeutic response. These configurations may influence sensitivity or resistance to chemotherapy, targeted therapies, and ICIs. Created with BioRender (https://app.biorender.com). Abbreviations: AKT, protein kinase B; AMPK, AMP-activated protein kinase; ATGs, autophagy-related genes; BECN1, Beclin 1; CD36, cluster of differentiation 36; CPT1A, carnitine palmitoyltransferase 1A; CRC, colorectal cancer; FAO, fatty acid oxidation; FASN, fatty acid synthase; GLS, glutaminase; GLUT1, glucose transporter 1; GPT2, glutamic pyruvate transaminase 2; HIF-1α, hypoxia-inducible factor 1 alpha; HK2, hexokinase 2; ICI, immune checkpoint inhibitor; IDO1/2, indoleamine 2,3-dioxygenase 1/2; IL, interleukin; LAT1, L-type amino acid transporter 1; LC3B, microtubule-associated protein 1 light chain 3 beta; LDHA, lactate dehydrogenase A; LPCAT1, lysophosphatidylcholine acyltransferase 1; MDSC, myeloid-derived suppressor cell; mTOR, mechanistic target of rapamycin; NDUFA4L2, NADH dehydrogenase 1 alpha subcomplex subunit 4-like 2; NK, natural killer; NO, nitric oxide; OXPHOS, oxidative phosphorylation; PDK4, pyruvate dehydrogenase kinase 4; PHGDH, phosphoglycerate dehydrogenase; PI3K, phosphoinositide 3-kinase; PKM2, pyruvate kinase M2; PPARGC1A, peroxisome proliferator-activated receptor gamma coactivator 1 alpha; PRKAA1, protein kinase AMP-activated catalytic subunit alpha 1; PSAT1, phosphoserine aminotransferase 1; PSPH, phosphoserine phosphatase; ROS, reactive oxygen species; TAM, tumor-associated macrophage; TGF-β, transforming growth factor beta; TME, tumor microenvironment; Tregs, regulatory T cells; VEGF, vascular endothelial growth factor.

Although the metabolic pathways discussed above are presented separately for clarity, they do not operate as independent processes in CRC. Rather, glycolysis, amino acid metabolism, lipid metabolism, mitochondrial function, autophagy, and endocrine metabolic signaling are interconnected components of a dynamic immunometabolic network. Through extensive metabolic crosstalk, these programs collectively regulate nutrient availability, redox balance, biosynthetic activity, and cellular adaptation to environmental stress. Importantly, the consequences of this metabolic reprogramming extend beyond tumor cells and profoundly influence the TME through nutrient competition and the accumulation of immunoregulatory metabolites such as lactate and kynurenine. These mechanisms promote the expansion and functional reprogramming of immunosuppressive cell populations, including regulatory T cells (Tregs), myeloid-derived suppressor cells (MDSCs), and tumor-associated macrophages (TAMs), while impairing the activity of cytotoxic lymphocytes. As illustrated in Figure 2, the convergence of these metabolic programs generates an integrated immunometabolic ecosystem that links tumor growth, immune suppression, metastatic potential, and resistance to chemotherapy, targeted therapies, and ICIs.

From a therapeutic perspective, these immunometabolic configurations are not merely descriptive metabolic phenotypes but may define distinct patterns of therapeutic vulnerability and resistance. Consequently, specific metabolic states could inform treatment selection by identifying tumors more likely to respond to metabolism-targeted interventions, immunotherapy combinations, or mitochondrial-directed approaches (Table 2). Glycolysis-dominant tumors are frequently associated with aggressive behavior, lactate-mediated immune suppression, and reduced sensitivity to chemotherapy. Adaptive states characterized by glutamine utilization, autophagy, and metabolic stress responses promote survival under therapeutic pressure and may contribute to resistance to conventional treatments. In contrast, lipid- and FAO-dependent configurations are increasingly linked to immune dysfunction and resistance to immune checkpoint blockade (ICB), suggesting that metabolic interventions combined with immunotherapy may represent a rational strategy for these tumors. Therefore, metabolic biomarkers may eventually contribute not only to prognostic stratification but also to the identification of therapeutically actionable metabolic states.

## 3. Therapeutic Strategies for CRC

The gold-standard therapy for early-stage (I–II) CRC is surgical resection of the primary tumor. In more advanced stages, in addition to surgery, patients receive adjuvant therapies, particularly those with metastatic disease. Conventional chemotherapy is based on multiple factors, including disease extent, metastatic pattern, patient performance status, comorbidities, and therapeutic goals (curative versus palliative intent). Equally important is a comprehensive molecular tumor characterization, which has become an essential prerequisite or optimal treatment planning [13,162].

### 3.1. Current Therapies

The first-line therapeutic approach in CRC is determined by tumor stage, molecular characteristics, treatment tolerability, and overall clinical context. In mCRC, a broad range of therapeutic options is available, administered either as monotherapy or in combination regimens [15]. The primary goals of first-line systemic therapy include prolonging OS, controlling tumor burden, alleviating symptoms, and, in selected cases, converting initially unresectable metastases into resectable disease. This phase of treatment is particularly critical, as it provides the highest response rates and the longest PFS, and represents the only therapeutic opportunity accessible to all patients. Consequently, the selection of the initial regimen has a substantial impact on subsequent treatment strategies and long-term clinical outcomes [13].

Standard first-line chemotherapy for CRC stages II (high-risk T3 and T4), III, and IV (with resectable metastases) relies on fluoropyrimidine-based regimens combined with additional agents (Table 3). The most widely used combinations include folinic acid, fluorouracil and oxaliplatin (FOLFOX) and capecitabine and oxaliplatin (CAPEOX). 5-FU and capecitabine, a prodrug of 5-FU, exert their antitumor effects primarily through inhibition of thymidylate synthase, whereas oxaliplatin is a platinum-based antineoplastic agent that acts as a “non-classical” or pseudo-alkylating agent which interferes with DNA replication and transcription through the formation of intra- and inter-strand cross-links [9,163]. Leucovorin enhances the efficacy of 5-FU by stabilizing its interaction with thymidylate synthase [164]. These regimens demonstrate comparable efficacy in terms of OS, and treatment selection is guided by toxicity profiles, prior therapies, and patient-specific considerations [165].

In stage IV CRC with unresectable metastases, systemic therapy is typically based on combination chemotherapy, often integrated with targeted agents (Table 3). Treatment selection is influenced by tumor-specific features, including primary tumor sidedness and key molecular alterations such as *RAS* and *BRAF* mutations. Right- and left-sided CRC differ substantially in embryologic origin, histopathology, carcinogenic pathways, and molecular profiles. Right-sided tumors are more frequently characterized by high MSI-H, CpG island methylator phenotype high, *BRAF* mutations, and increased immune cell infiltration. In contrast, left-sided tumors more commonly exhibit chromosomal instability, *Adenomatous polyposis coli* (*APC*) and *TP53* mutations, EGFR pathway alterations, and *HER2* amplification. These biological differences translate into distinct clinical behaviors, as patients with right-sided tumors generally experience poorer outcomes and reduced OS in stage III and metastatic disease compared with those with left-sided tumors [166]. Activating *RAS* and *BRAF* mutations occur in approximately 45% and 10% of CRC cases, respectively [167,168]. Constitutive activation of these oncogenes leads to persistent MAPK pathway signaling, driving uncontrolled proliferation, tumor progression, and resistance to therapy. Because the RAS/BRAF axis functions downstream of the EGFR, tumors harboring these mutations do not benefit from EGFR-targeted therapies [169].

The integration of targeted therapies has significantly improved outcomes in mCRC and represents a central element of first-line treatment. Accordingly, management of unresectable mCRC is based on combination chemotherapy, frequently combined with targeted agents (Table 3). In patients with left-sided, *RAS/BRAF* wild-type tumors and unresectable synchronous liver or lung metastases, first-line treatment typically includes FOLFOX or folinic acid, 5-FU and irinotecan (FOLFIRI) in combination with anti-EGFR monoclonal antibodies (mAbs) such as cetuximab or panitumumab. A similar approach is adopted in patients with metachronous metastases, although irinotecan-based regimens are often preferred in this setting. Irinotecan is a prodrug converted into its active metabolite SN-38, which inhibits topoisomerase I, while cetuximab and panitumumab specifically target EGFR [170,171]. In contrast, patients harboring *RAS* or *BRAF* mutations are generally treated with chemotherapy backbones such as FOLFOX, FOLFIRI, CAPEOX, or folinic acid, 5-FU, irinotecan and oxaliplatin (FOLFOXIRI), administered either alone or in combination with anti-angiogenic agents. Bevacizumab targets VEGF-A and ziv-aflibercept is a recombinant fusion protein that acts as a potent decoy receptor, targeting VEGF-A, VEGF-B, and placental growth factor, whereas ramucirumab targets VEGF receptor 2 (VEGFR2) [9]. Treatment selection further depends on disease timing and prior therapy. Patients with metachronous metastatic disease may receive irinotecan-based regimens alone or in combination with VEGF pathway inhibitors, while oxaliplatin-based combinations can be reintroduced after prior irinotecan exposure in selected cases.

Bevacizumab can be combined with all standard chemotherapy backbones and is applicable across molecular subtypes. Its addition to first-line chemotherapy provides consistent improvements in PFS and modest gains in OS [172]. Anti-EGFR mAb, including cetuximab and panitumumab, are restricted to patients with RAS wild-type tumors [13]. Extensive evidence indicates that RAS mutations are strong negative predictive biomarkers for EGFR-targeted therapy, precluding clinical benefit in this subgroup [15].

Primary tumor sidedness further refines therapeutic decision-making in mCRC. Patients with left-sided, RAS wild-type tumors derive a clear benefit from EGFR-targeted therapy in the first-line setting, whereas those with right-sided tumors generally do not, despite occasional improvements in response rates. This differential efficacy reflects underlying biological differences that influence treatment sensitivity. Combination regimens may increase toxicity and should therefore be reserved for carefully selected patients. The treatment strategies outlined above are primarily applicable to patients with pMMR/MSS tumors. Additional details are available in the National Comprehensive Cancer Network (NCCN) Guidelines (NCCN.org).

A distinct subset of mCRC cases (approximately 3.5–5%), more frequently arising in right-sided tumors, is characterized by dMMR/MSI-H [15]. These tumors display marked sensitivity to ICIs, which have become a key component of first-line treatment in this setting. In patients with stage II (T4b) and stage III disease, fluoropyrimidine-based chemotherapy regimens such as FOLFOX or CAPEOX may be administered either alone or in combination with ICIs, including atezolizumab, a mAb targeting programmed death ligand 1 (PD-L1) [173]. Patients with stage IV disease and resectable synchronous metastases may undergo surgical resection followed by adjuvant chemotherapy with FOLFOX or CAPEOX. Alternatively, neoadjuvant immunotherapy may be administered before surgery, allowing both primary tumor and metastatic lesions to be resected in selected cases. In the presence of unresectable metastatic disease, ICIs represent the favored therapeutic approach. Nivolumab, an anti-PD-1 receptor mAb, is commonly used either as monotherapy or in combination with ipilimumab, an anti-cytotoxic T-lymphocyte-associated antigen 4 (CTLA-4) mAb; similarly, pembrolizumab (also targeting PD-1) is frequently employed as monotherapy in this setting [173]. These therapeutic strategies reflect the high immunogenicity of MSI-H/dMMR tumors and underscore the importance of integrating immunotherapy into treatment algorithms for this biologically distinct CRC subgroup. Comprehensive recommendations are available in the NCCN Guidelines (NCCN.org).

ICIs have substantially improved clinical outcomes in patients with MSI-H/dMMR mCRC, establishing immunotherapy as a cornerstone of treatment in this molecularly defined subgroup. These advances highlight the necessity of comprehensive molecular profiling, which is essential to identify patients most likely to benefit from immune-based strategies and for guiding therapeutic decision-making. Treatment options for MSI-H/dMMR mCRC are summarized in Supplementary Table S1.

Second-line therapy is indicated for patients with mCRC who experience disease progression after first-line treatment and aims to prolong survival, control symptoms, and preserve quality of life. Treatment selection is highly individualized and depends on prior therapy, molecular profile, tumor sidedness, performance status, and treatment-related toxicities [15,174]. In most cases, management involves switching the chemotherapy backbone and integrating targeted agents according to prior exposure and tolerability. Following progression on oxaliplatin-based regimens (FOLFOX or CAPEOX), irinotecan-based therapy (FOLFIRI) represents the standard second-line option. In *RAS/BRAF* wild-type tumors, irinotecan-containing regimens are typically combined with anti-EGFR agents, whereas in tumors harboring *RAS* or *BRAF* mutations, treatment relies on irinotecan-based therapy alone or in combination with VEGF pathway inhibitors. Conversely, oxaliplatin-based combinations may be reintroduced after irinotecan failure in selected patients. When both oxaliplatin and irinotecan have been previously administered, subsequent strategies are guided by molecular status and prior exposure. In *RAS/BRAF* wild-type disease, irinotecan alone or combined with anti-EGFR agents remains a preferred approach. In contrast, patients with *RAS/BRAF*-mutated tumors may receive FOLFOX or CAPEOX, either alone or with bevacizumab, as well as irinotecan-based regimens combined with VEGF inhibitors, including bevacizumab, ziv-aflibercept or ramucirumab. More intensive combinations, such as FOLFOXIRI with or without bevacizumab, may be considered in selected cases. Detailed treatment algorithms are provided in the NCCN Guidelines (NCCN.org).

Selected molecular subgroups may benefit from dedicated targeted strategies in later treatment lines. Patients harboring the *BRAF* V600E mutation, *HER2* amplification, or *Neurotrophic Tyrosine Receptor Kinase* (*NTRK*) gene fusions may receive specific targeted therapies [175,176]. In patients with dMMR/MSI-H, ICIs remain an effective option even after prior systemic therapy. In cases of prior exposure to ICI monotherapy, a combination of nivolumab and ipilimumab is preferred. An overview of second-line therapeutic options is provided in Supplementary Table S2.

### 3.2. New Drugs in Progress

Despite significant progress, CRC remains a leading cause of cancer-related mortality worldwide, mainly due to the development of metastatic disease and the biological heterogeneity that limits uniform treatment benefit [177]. To enhance therapeutic efficacy, one of the main contemporary goals is the implementation of biomarker-matched approaches and strategies aimed at converting “immunologically cold” MSS tumors into responsive disease. Indeed, MSS CRC accounts for the most metastatic cases and is typically resistant to PD-1/PD-L1 monotherapy, making it a central focus of experimental immunotherapy research [178].

Here, we describe the most relevant clinical trials currently ongoing or recently completed, with potential implications for the development of novel drugs and combination strategies in CRC, emphasizing mechanistic rationale, study design, and translational considerations. As previously mentioned, several studies aim to convert “cold” MSS tumors into a “hot” immune microenvironment that may respond to ICIs, particularly when combined with chemotherapy or targeted agents.

Several phase I and II clinical trials are ongoing or have recently been completed (Table 4). In particular, preliminary findings from combinations such as pembrolizumab plus CAPOX plus bevacizumab (NCT04262687), sintilimab plus regorafenib (NCT04745130), and regorafenib plus ipilimumab plus nivolumab (NCT04362839) have shown encouraging clinical activity, supporting further evaluation in subsequent trial phases [179,180,181,182]. Moreover, additional trials investigating novel ICI agents, such as botensilimab (targeting CTLA-4) and balstilimab (targeting PD-1), administered either as monotherapy or in combination and specifically designed for MSS CRC, are currently ongoing (NCT03860272, NCT05571293, NCT07152821, and NCT05608044). Preliminary results suggest that the combination of these ICIs provides significant and durable responses in heavily pretreated MSS tumors compared with monotherapy or standard-of-care treatments, particularly in patients without active liver metastases [183,184,185].

Interestingly, the STELLAR-303 study (NCT05425940), which evaluated the multi-target tyrosine kinase inhibitor (TKI) zanzalintinib in combination with atezolizumab (targeting PD-L1), significantly improved OS compared with regorafenib in patients with MSS mCRC refractory to prior treatments. These data indicate that STELLAR-303 represents the first phase III immunotherapy-based study to demonstrate a survival benefit in this setting. Therefore, the combination of zanzalintinib plus atezolizumab may potentially represent a new standard of care for refractory MSS mCRC [186,187].

To further enhance the efficacy of immunotherapy in dMMR/MSI-H CRC patients, several clinical trials investigating next-generation ICIs or combinations of currently available ICIs with chemotherapy and targeted agents have been designed (Table 4). Treatment with nivolumab plus ipilimumab (NCT04008030) improved patient outcomes compared with nivolumab monotherapy [188,189]. Moreover, combinations such as mFOLFOX6 plus bevacizumab plus atezolizumab (NCT02997228) and mFOLFOX6 plus atezolizumab (NCT02912559) were associated with increased PFS and a reduced risk of disease recurrence, respectively [190,191]. Novel ICIs, including dostarlimab and cemiplimab (anti-PD-1) and ivonescimab (a first-in-class bispecific Ab that simultaneously targets PD-1 and VEGF), are currently being evaluated in several ongoing clinical trials (NCT05723562, NCT04165772, NCT05855200, NCT05961709, and NCT06959550). Treatment with dostarlimab has demonstrated high rates of clinical complete response and has enabled nonoperative management in patients with dMMR CRC. However, results from most of these trials are not yet available [192,193,194].

Additional clinical trials focus on CRC harboring mutations in *KRAS*, *BRAF*, and *HER2*, with particular attention to the *BRAF* V600E mutation, which is associated with poor prognosis (Table 4). To improve OS in these patients, several studies are evaluating the BRAF inhibitor encorafenib in combination with cetuximab and ICIs, with or without chemotherapy (NCT05217446, NCT04607421, NCT04017650, and NCT03388190). Preliminary data suggest that these combinations may synergistically enhance antitumor activity, leading to improvements in PFS and OS in patients with *BRAF* V600E-mutated CRC [195,196,197,198,199].

*KRAS* mutations are among the most common activating mutations in CRC, occurring in approximately 40–50% of cases. Among these mutations affecting codon G12 are particularly relevant, although specific variants (e.g., G12C) occur in a smaller proportion of patients. Some *KRAS* mutations have been associated with worse prognosis compared with *KRAS* wild-type tumors or tumors harboring other KRAS variants [200]. Consequently, several clinical trials are evaluating KRAS inhibitors, administered either as monotherapy or in combination with EGFR inhibitors (NCT05198934, NCT04793958, NCT03785249, NCT04449874, NCT07020221, and NCT06917079). Preliminary results indicate improvements in PFS, OS, and overall antitumor activity compared with standard therapies [200,201,202,203,204].

*HER2* amplification is detected in approximately 3–4% of mCRC cases. Several clinical trials have been designed to improve survival and quality of life in this patient population. The combination of tucatinib and trastuzumab demonstrated promising antitumor activity in a phase II clinical trial (NCT03043313) [205], with phase III data are still pending (NCT05253651) [11]. Similarly, trastuzumab deruxtecan has shown encouraging antitumor activity with a favorable safety profile (NCT04744831) [206].

Other next-generation therapeutic strategies (Table 4) include antibody-drug conjugates such as sacituzumab govitecan and telisotuzumab adizutecan, as well as the bispecific antibody amivantamab targeting EGFR and MET receptors. These agents are being evaluated either alone or in combination with other therapies in ongoing clinical trials enrolling mCRC patients (NCT06243393, NCT05379595, NCT06750094, and NCT07023289). As these studies are still ongoing, only trial design and preliminary information are currently available [207].

Finally, the umbrella trial (NCT04929223) adopts an umbrella design to evaluate multiple experimental targeted therapies and immunotherapy combinations tailored to the biomarker profile of each mCRC patient (Table 4). This study tests several investigational regimens in parallel cohorts stratified according to specific genetic or molecular signatures. Such a design enables the evaluation of personalized therapeutic strategies within a single overarching protocol. The trial is currently ongoing, and results are not yet available.

Taken together, the data described above suggest that combinations including anti-VEGF TKIs, novel immunomodulatory agents, and BRAF or KRAS inhibitors are effective therapeutic strategies. These findings are particularly encouraging, as results obtained with the combination of standard chemotherapy and ICIs in the first-line setting have shown modest benefits, especially in MSS tumors [208]. Further research is needed to define the most effective therapeutic strategies for patients with metastatic CRC.

## 4. Obesity and Adipose Tissue-Driven Immunometabolic Dysfunction in CRC

### 4.1. Adipose Tissue as a Systemic Immunometabolic Driver

Adipose tissue is now recognized as a dynamic endocrine and immunometabolic organ that contributes to CRC progression through inflammatory signaling, metabolic rewiring, and adipokine-mediated effects [209]. In obesity, adipocyte dysfunction sustains chronic low-grade inflammation, marked by increased interleukin-6 (IL-6), Tumor Necrosis factor (TNF)-α, and IL-1β, which activate pro-tumorigenic pathways such as Nuclear factor kappa B (NF-κB) and signal transducer and activator of transcription 3 (STAT3) in epithelial and stromal compartments. Concurrently, hyperinsulinemia and activation of the insulin/IGF-1 axis promote proliferative and anti-apoptotic signaling, fostering tumor growth and survival. Adipose tissue also secretes adipokines with divergent functions, including leptin and adiponectin, which influence tumor behavior through Janus kinase (JAK)/STAT, PI3K/AKT, and AMPK pathways [210]. In parallel, enhanced lipolysis in dysfunctional adipose tissue increases the release of free FAs (FFAs), which provide substrates for tumor proliferation and metabolic adaptation [211]. Together, these adipose tissue-derived factors act as central regulators of metabolic and immune processes linking obesity-associated inflammation to CRC development, progression, and recurrence [212].

Visceral obesity is a major determinant of CRC risk because visceral adipose tissue (VAT) exhibits a more metabolically active and pro-inflammatory phenotype than subcutaneous fat [213]. It is associated with activation of oncogenic pathways, including NF-κB and STAT3, that sustain tumor initiation and progression. Visceral adiposity is also tightly linked to insulin resistance and hyperinsulinemia, leading to activation of the insulin/IGF-1 axis and downstream PI3K/AKT and MAPK signaling, thereby promoting proliferation and inhibiting apoptosis. In addition, dysregulated adipokine secretion and increased lipolytic activity in VAT raise circulating FFAs, which tumor cells exploit to support metabolic adaptation and growth. Beyond these metabolic effects, visceral obesity is associated with gut microbiota dysbiosis and increased intestinal permeability, facilitating the translocation of bacterial products such as lipopolysaccharide (LPS) and sustaining mucosal inflammation [214]. Visceral adiposity, therefore, integrates metabolic, inflammatory, and microbiota-driven mechanisms that converge to promote CRC development and progression [215,216].

The link between metabolic disorders and cancer is driven largely by insulin resistance, which establishes a systemic pro-tumorigenic milieu [217]. Hyperglycemia and insulin resistance sustain chronic inflammation, reinforce oncogenic signaling in the intestinal epithelium, particularly through NF-κB and STAT3, and compromise barrier integrity, thereby facilitating microbial translocation. In parallel, diabetes promotes immune remodeling characterized by reduced cytotoxic T-cell activity and expansion of immunosuppressive myeloid populations, weakening immune surveillance. These effects are further amplified by diabetes-associated dysbiosis, which increases the production of pro-inflammatory metabolites and microbial components, including secondary bile acids and endotoxins. At the same time, hyperinsulinemia, IGF-1 activation, and oxidative stress converge with microbiota-derived cues to sustain epithelial oncogenic pathways. Together, these processes establish a metabolically and immunologically permissive microenvironment that promotes colorectal tumorigenesis [218].

#### 4.1.1. Diet–Microbiota–Inflammasome Axis in Obesity-Associated CRC

Dietary patterns and obesity-associated dysbiosis reinforce the role of adipose tissue as an immunometabolic hub. The gut microbiome modulates metabolic outputs, epithelial integrity, and immune responses in CRC [219]. Obesity-related dysbiosis alters microbial metabolite production, reducing Short-chain FAs (SCFAs) and increasing pro-inflammatory and genotoxic compounds, thereby promoting epithelial stress and tumorigenic signaling [220]. These changes are accompanied by impaired barrier function and increased translocation of microbial components, sustaining chronic immune activation and a pro-tumorigenic microenvironment [220].

Innate immune sensing pathways link microbial signals to sustained inflammatory responses. In particular, engagement of pattern recognition receptors such as Toll-like receptor 4 (TLR4) by microbiota-derived components, including LPS, promotes NF-κB-dependent priming of the NLR family pyrin domain containing 3 (NLRP3) inflammasome, leading to the maturation and release of IL-1β and IL-18 [221]. This axis amplifies epithelial stress, impairs barrier integrity, and perpetuates inflammatory responses that support tumor-promoting pathways. Sustained inflammasome activation also contributes to immune dysregulation by fostering myeloid-driven inflammation and limiting effective antitumor immunity [222]. The TLR4–NLRP3 inflammasome axis therefore represents a central mechanistic node through which microbiota-derived signals, metabolic dysfunction, and immune remodeling converge to promote colorectal tumorigenesis, and it may also represent a therapeutic target [222].

#### 4.1.2. Adipocyte–Tumor Crosstalk and Metabolic Reprogramming

Adipocytes and tumor cells exchange metabolic signals that shape local behavior. Integrative transcriptomic analyses from murine models and the ColoCare patient cohort indicate that obesity induces conserved inflammatory and metabolic gene expression programs within colon tumors, including upregulation of immune-related genes such as IL6, CXCL8 (IL-8), and CCL2, as well as pathways involved in metabolic adaptation and lipid handling [223]. Network analyses further reveal enhanced ligand–receptor interactions between adipose tissue and tumor cells, particularly along chemokine and cytokine signaling axes that promote immune-cell recruitment and microenvironmental remodeling. Obesity-associated transcriptional changes also include increased expression of genes involved in extracellular matrix remodeling and tumor progression, such as MMP9 and CD44, further supporting the contribution of adipose-derived signals to invasive tumor phenotypes. Together, these findings indicate that obesity-driven transcriptional rewiring extends beyond systemic effects and reflects active, bidirectional communication between adipose tissue and tumor cells that drives CRC progression.

This interaction also involves strong metabolic coupling. Adipose depots adjacent to the tumor undergo extensive remodeling, including enhanced lipolysis, fibrosis, partial browning, and dedifferentiation into cancer-associated adipocytes (CAAs). These changes promote the release of FAs and other metabolic substrates that fuel tumor growth and support glycolytic reprogramming, EMT, and invasive behavior, underscoring the role of adipose tissue as an active metabolic partner [224]. Lipid transfer further sustains this interaction, as FA transporters such as CD36 [225,226] and lipid chaperones including FA-binding protein 4 [227] facilitate the uptake and utilization of adipocyte-derived lipids by tumor cells, thereby enhancing energy production and metastatic potential.

#### 4.1.3. TME Remodeling and Intercellular Communication

Hypoxia is a defining feature of dysfunctional adipose tissue in obesity [228,229] and a central driver of adipocyte–tumor crosstalk in CRC. As adipose depots expand, inadequate vascularization reduces oxygen availability and stabilizes HIF-1α, thereby inducing transcriptional programs associated with inflammation, angiogenesis, and metabolic adaptation. In adipocytes, HIF-1α activation promotes the secretion of pro-inflammatory cytokines, including IL-6 and TNF-α, as well as adipokines and extracellular matrix components, reinforcing a tumor-supportive microenvironment [230].

Hypoxia-driven signaling also directly influences tumor cell behavior by enhancing glycolytic flux, angiogenic pathways (e.g., VEGF), and EMT, thereby facilitating tumor progression and adaptation to nutrient-deprived conditions. In parallel, hypoxic adipose tissue promotes immune dysfunction by recruiting immunosuppressive cell populations and reshaping cytokine gradients, further linking metabolic stress to tumor-promoting inflammation. This crosstalk also extends to extracellular matrix remodeling and increased tissue stiffness within peritumoral adipose depots, characterized by enhanced collagen deposition and activation of Transforming growth factor beta (TGF-β)-related pathways that promote tumor invasion and microenvironmental reorganization [231].

Peritumoral adipose tissue also contributes to immune evasion. Adipose-derived stromal cells modulate immune-cell trafficking and function, in part through the CXCL12–CXCR4 signaling axis, leading to sequestration or exclusion of effector lymphocytes from the TME. These stromal cells may acquire fibroblast-like features and give rise to adipose-derived cancer-associated fibroblasts, thereby reinforcing an immunosuppressive, tumor-promoting niche [232].

Extracellular vesicles and microRNAs further mediate adipocyte–tumor crosstalk by enabling fine-tuned epigenetic regulation of tumor cells. Adipocyte-derived exosomes transfer specific miRNAs to CRC cells, including miR-21, miR-155, and miR-34a, thereby modulating gene-expression programs involved in proliferation, invasion, and metabolic adaptation. MiR-21 and miR-155 are associated with pro-inflammatory and pro-tumorigenic signaling that enhances NF-κB activation and EMT, whereas miR-34a, although classically considered tumor-suppressive, has also been implicated in metabolic adaptation and adipocyte dysfunction in obesity. These miRNA-mediated interactions reshape pathways related to inflammation, lipid metabolism, and tumor progression, thereby reinforcing tumor-promoting phenotypes. Tumor-derived signals can, in turn, reprogram the miRNA profile of adipocytes, contributing to their dedifferentiation and conversion into CAAs. This bidirectional exchange of non-coding RNAs adds another regulatory layer to adipose-tumor communication and has potential implications for biomarker development and therapeutic targeting [233]. Several of these miRNAs are also detectable in circulation, supporting their potential use as minimally invasive biomarkers.

#### 4.1.4. Adipokine Signaling and Systemic Inflammation

Adipokines provide clinically measurable indicators of the systemic metabolic imbalance linking obesity to CRC. Rather than supporting a strict dichotomy, current evidence points to a context-dependent imbalance between pro-tumorigenic adipokines, such as leptin and resistin, and protective mediators such as adiponectin [234]. Leptin and resistin promote tumorigenic processes via JAK/STAT, PI3K/AKT, and NF-κB signaling, thereby enhancing tumor growth, angiogenesis, and immune modulation, whereas adiponectin exerts protective effects through AMPK-dependent pathways and anti-inflammatory signaling.

Meta-analytic evidence more consistently links reduced adiponectin levels to CRC risk and progression, whereas the relationship between circulating leptin and CRC remains heterogeneous and appears influenced by factors such as sex, fat distribution, and disease stage. The relative balance between pro- and anti-tumor adipokines, rather than the absolute level of any single molecule, therefore, emerges as a critical determinant of CRC risk and progression [235].

This imbalance operates in concert with inflammatory mediators derived from adipose tissue. TNFα and leptin, in particular, act synergistically to promote colorectal tumor growth, reinforcing a pro-inflammatory microenvironment and amplifying tumor-supportive signaling networks [236]. This interplay between adipokines and inflammatory mediators illustrates how systemic metabolic dysfunction is translated into tumor-promoting cues within the CRC microenvironment. Adipose tissue, therefore, emerges as a central endocrine and immunometabolic hub in CRC, integrating chronic inflammation, adipokine imbalance, insulin resistance, lipid mobilization, microbiota-driven signaling, and direct adipocyte–tumor crosstalk into a coordinated network that sustains tumor initiation, progression, and dissemination.

### 4.2. Visceral Obesity as a Major Risk Determinant of CRC

Visceral obesity is a biologically distinct and clinically relevant risk factor for CRC. Early risk-stratification strategies for advanced colorectal neoplasia relied largely on body mass index (BMI)-based models [237]. A large-scale systematic analysis conducted in the United States between 2000 and 2019, which included CRC among BMI-associated malignancies, evaluated cause-specific years of life lost (YLLs) attributable to non-optimal BMI across counties, stratified by sex, race, and ethnicity. The study showed that elevated BMI contributes substantially to premature mortality, with a measurable proportion of CRC-related YLLs attributable to excess adiposity. Notably, the burden of BMI-associated CRC mortality showed marked geographic and demographic variation, with higher YLL rates in regions with greater obesity prevalence and among specific racial and ethnic groups. These findings reinforce the role of obesity as a modifiable CRC risk factor and highlight persistent disparities in its impact across populations.

A large, pooled cohort study from the Asia Cohort Consortium, comprising more than 600,000 participants, examined the association between BMI and CRC incidence and mortality in Asian populations. Individuals with a BMI > 30.0 showed a significantly increased risk of CRC incidence and CRC-related mortality, with a clear dose–response relationship compared with those within the reference range. The association was stronger for colon cancer than for rectal cancer and more pronounced in men [238]. Prospective meta-analyses are consistent with these findings and report a dose-dependent increase in CRC risk with rising BMI, with stronger effects in colon cancer and in male individuals [239].

However, the anthropometric measures most closely associated with CRC risk remain incompletely defined. A large cohort study including approximately 458,000 participants from the UK Biobank compared BMI, as a measure of overall adiposity, with waist circumference (WC) and waist-to-hip ratio (WHR), which more accurately reflect central fat distribution [214]. WC and WHR showed stronger associations with CRC incidence than BMI, indicating that reliance on BMI alone may underestimate the contribution of excess adiposity to tumorigenesis.

These observations are further supported by studies using direct imaging-based measures of visceral adiposity. A retrospective study evaluated whether quantitative computed tomography (CT)-derived parameters, rather than BMI alone, could predict short-term postoperative outcomes in one hundred patients undergoing colectomy for stage I-III colon cancer. Visceral obesity, defined by visceral fat area (VFA) and visceral-to-subcutaneous fat ratio (V/S), was associated with a higher prevalence of metabolic comorbidities, including hypertension and diabetes, whereas BMI-based classification showed no such associations [240]. Elevated VFA and, to a lesser extent, V/S were also linked to significantly higher rates of postoperative complications, whereas BMI was not associated with increased morbidity. These findings indicate that CT-derived measures of visceral adiposity identify patients at increased risk for postoperative complications more accurately and support their use in preoperative risk stratification for colon cancer.

The association between adiposity and CRC risk appears to be sex-dependent, reflecting distinct contributions of overall and central obesity in men and women. In a cohort of approximately 287,000 German individuals, overweight and obesity were associated with an increased risk of gastrointestinal cancers, including CRC. However, these associations were stronger and more consistent in men, whereas findings in women varied by cancer type [241].

A Mendelian randomization study further examined the causal relationship between obesity, fat distribution, and cancer risk using genetic variants as proxies for BMI and central adiposity measures, including WHR. By leveraging large-scale genetic datasets, this approach reduces confounding and reverse causation inherent in observational studies. The analysis showed that genetically predicted BMI was more strongly associated with CRC risk in women, whereas central adiposity, as reflected by WHR, was more strongly associated in men. These findings indicate that both the amount and distribution of adiposity are critical determinants of CRC risk and support the existence of sex-specific biological mechanisms underlying these associations [242]. These studies consistently indicate that measures of visceral adiposity outperform BMI in capturing CRC-associated metabolic risk, although the relative contribution of sex-specific hormonal, genetic, and metabolic determinants remains incompletely understood.

In women, the relationship between visceral obesity and CRC risk is further influenced by endogenous sex hormones, particularly in the postmenopausal setting. A cohort of approximately 1000 European subjects evaluated circulating levels of estrogens, androgens, progesterone, and sex hormone-binding globulin, but did not identify clear associations between most hormone levels and CRC risk. These data indicate that the protective effects of hormone therapy are not explained solely by circulating hormone concentrations [243].

The role of estrogen in obesity-associated tumor development remains complex and context-dependent. Although obesity supports tumor development through metabolic and inflammatory mechanisms, estrogen signaling may exert both protective and pro-tumorigenic effects. This apparent discrepancy may be explained in part by the differential roles of estrogen receptor subtypes: estrogen receptor alpha (ERα) is associated with proliferative signaling, whereas estrogen receptor beta (ERβ), which is predominantly expressed in normal colonic epithelium, exerts anti-proliferative and pro-apoptotic effects. During tumor progression, a shift from ERβ to ERα expression may occur, potentially converting estrogen signaling from protective to tumor-promoting. This receptor-specific balance may underlie inconsistent epidemiological findings and represents a potential target for prevention and therapeutic intervention [244].

Environmental endocrine disruptors further modulate the relationship between visceral obesity and CRC. Bisphenol A (BPA), a xenoestrogen, can bind classical estrogen receptors (ERs) and activate non-classical signaling pathways, including membrane-associated receptors such as G protein-coupled estrogen receptor (GPER), thereby influencing genomic and non-genomic responses, including MAPK/ERK, PI3K/AKT, and PKA signaling [245]. In the colorectal context, BPA disrupts the balance between ERα and ERβ signaling. Although ERβ generally exerts protective effects in the colonic epithelium, BPA may antagonize some of its functions while promoting pro-tumorigenic pathways, including ERK activation, downregulation of E-cadherin, and upregulation of EMT markers such as Snail, N-cadherin, and vimentin. These alterations promote proliferation, motility, invasion, and resistance to apoptosis [246].

Experimental evidence from animal models further suggests that BPA exposure may exacerbate CRC progression [247], particularly in the context of obesity, where it is associated with worsened histopathological features, increased inflammatory signaling, and systemic alterations without significantly affecting body weight. These effects have been linked to pathways involved in tumor progression, including PI3K/AKT signaling. BPA therefore emerges as a potential modulator of obesity-associated CRC susceptibility and development, although further studies are needed to clarify its sex-specific and receptor-mediated effects.

Evidence on GPER expression further highlights the complexity of estrogen signaling in the gastrointestinal tract. A study investigating GPER expression in gastric and colonic smooth muscle of male and female non-obese diabetic (NOD) mice demonstrated sex-dependent differences associated with histone modifications, particularly H3K4me3 and H3K27ac [248]. Taken together, estrogen signaling may be dynamically regulated at the epigenetic level, contributing to sex-related variability in receptor expression and activity. Such mechanisms are particularly relevant in the context of endocrine disruptor exposure, including BPA, and may help explain the context-dependent and sex-specific effects observed during CRC pathogenesis. Epigenetic modulation of GPER and other estrogen-related receptors may therefore represent a key determinant of differential susceptibility to obesity-associated CRC and of heterogeneous responses to endocrine disruptors, warranting further investigation.

A bioinformatic analysis further explored the molecular links between obesity and colon cancer, with a specific focus on female patients. Integration of gene-expression datasets identified 146 differentially expressed genes (DEGs) shared between obesity and colon cancer [249]. Functional enrichment analysis indicated that these genes are involved predominantly in inflammatory and immune-related pathways. Protein–protein interaction network analysis further highlighted a subset of hub genes, including CD44, CXCR4, IL6, MMP9, and IGF1, suggesting their central role in obesity-associated tumor-promoting networks. These genes also appear to be regulated by key transcription factors and microRNAs, pointing to a multilayered regulatory framework linking metabolic dysfunction.

Visceral obesity should therefore be regarded not simply as excess body fat, but as a biologically distinct condition associated with increased CRC risk and adverse clinical outcomes. Compared with BMI alone, measures of central and visceral adiposity more accurately capture the pathogenic fat distribution underlying colorectal tumorigenesis. This relationship is further shaped by sex-specific hormonal and receptor-dependent mechanisms, as well as by endocrine-disrupting exposures and obesity-associated inflammatory signaling networks. Within this framework, visceral obesity emerges as a major determinant of CRC risk, integrating anthropometric, metabolic, hormonal, and molecular dimensions into a unified biological model.

### 4.3. Metabolic Dysregulation as Systemic Drivers of CRC Risk and Tumorigenesis

#### 4.3.1. Metabolic Syndrome and Diabetes

Diabetogenic processes underlying insulin resistance-associated hyperinsulinemia are increasingly linked to CRC [217], supporting a direct contribution of metabolic dysregulation to tumorigenesis [250]. A comprehensive analysis conducted in China from 1990 to 2021 indicates that metabolic dysfunction has progressively contributed to the growing burden of CRC. Type 2 diabetes mellitus (T2DM) and elevated fasting glucose emerged as major drivers, with a substantial increase in disability-adjusted life years over time. The study also reported higher incidence rates in men and older individuals, alongside concerning upward trends in younger populations. These findings highlight a sustained increase in CRC incidence associated with metabolic risk factors, paralleling rapid lifestyle and socioeconomic changes, and underscore the need for targeted metabolic prevention strategies [251].

Yau et al. examined how multiple clinical and demographic factors interact to influence CRC risk in individuals with T2DM, adopting an integrative rather than single-factor approach [252]. Using a survival tree model, age emerged as the primary determinant of CRC risk, with individuals aged ≥65 years representing the highest-risk group. In younger patients, CRC risk was strongly influenced by metabolic control and disease duration, as poor glycemic control and longer-standing T2DM were associated with markedly increased risk. Additional factors, including male sex and comorbidities such as cardiovascular disease, further contributed to risk stratification. These findings indicate that CRC risk in T2DM arises from complex, non-linear interactions among clinical and metabolic variables rather than from individual risk factors alone.

Further evidence supports metabolic dysfunction as a key determinant of CRC initiation and progression, including at earlier ages [253]. Metabolic syndrome and its associated comorbidities are significantly linked to an increased risk of early-onset colorectal cancer (EOCRC). Among its components, T2DM, obesity, hypertension, and dyslipidemia all contribute to elevated risk, with the strongest effects generally attributed to diabetes and excess adiposity. CRC risk increases with the number of metabolic abnormalities, indicating a cumulative, potentially synergistic effect. These findings reinforce that not only the presence of T2DM, but also the broader context and severity of metabolic imbalance, critically shape CRC risk across age groups, including individuals traditionally considered at lower risk. Emerging evidence further suggests that EOCRC may, at least in part, represent a metabolically driven disease rather than solely an age-related condition [254]. Individuals with EOCRC frequently exhibit metabolic abnormalities, including obesity, insulin resistance, and T2DM, defining a clinical and biological profile distinct from that of traditional, age-related CRC. Real-world data from large population-based cohorts indicate that younger CRC patients often present with a higher burden of metabolic comorbidities [255], reinforcing the contribution of systemic metabolic alterations to early tumor development. These observations support a paradigm shift in which EOCRC is increasingly viewed as a manifestation of underlying metabolic dysfunction, highlighting the need for risk stratification strategies that extend beyond chronological age.

The extent and progression of T2DM, rather than the diagnosis alone, are key determinants of colorectal carcinogenesis and clinical outcomes [256]. A machine learning-based analysis of 10,749 CRC patients with T2DM demonstrated improved predictive accuracy for OS compared with conventional models. By integrating variables such as age, tumor stage, comorbidity burden, and metabolic status, these models enabled more precise risk stratification and outcome prediction. These findings highlight the value of integrative approaches in capturing the complex interplay between clinical and metabolic factors and improving prognostic assessment in CRC patients with T2DM [257].

Several interconnected biological mechanisms underpin the link between metabolic dysfunction and tumorigenesis. Chronic hyperinsulinemia and insulin resistance enhance insulin and IGF-1 signaling, promoting cell proliferation and inhibiting apoptosis. These effects are accompanied by persistent low-grade inflammation and alterations in adipokine profiles, which further sustain tumor-promoting pathways. Hyperglycemia contributes to tumor development by providing metabolic substrates for cancer cells and inducing oxidative stress [258]. These processes establish a pro-tumorigenic environment characterized by sustained proliferative, inflammatory, and anti-apoptotic signaling.

#### 4.3.2. Metabolic Modulators and Therapeutic Implications

Therapeutic strategies targeting insulin signaling and metabolic homeostasis, including incretin-based therapies, are increasingly investigated as modulators of CRC risk. Glucagon-like peptide-1 receptor agonists (GLP-1RAs), widely used in the treatment of type 2 diabetes and obesity, reduce hyperinsulinemia and improve insulin sensitivity, while exerting indirect effects on inflammatory and metabolic pathways implicated in tumor progression, including the IGF-1 and PI3K/AKT axes.

Mendelian randomization analyses have suggested a potential increase in CRC risk associated with GLP-1 receptor activation [259]; however, these findings have not been consistently supported by clinical or real-world data and should be interpreted with caution [260]. In contrast, observational studies generally report a neutral or potentially reduced CRC risk associated with GLP-1RAs [261,262,263].

Mechanistically, GLP-1RAs may act as host-directed modulators of the endocrine–metabolic axis, influencing systemic metabolism, inflammation, and immune responses. Through these effects, they may contribute to reshaping the TME and potentially enhancing responses to ICIs, supporting their role as adjunctive modulators in CRC within an integrated immuno-oncological framework [264].

Metformin is also considered a potential metabolic modulator, improving insulin sensitivity and reducing hyperinsulinemia, while exerting additional effects through inhibition of mitochondrial complex I and activation of AMPK. Through these mechanisms, metformin may counteract key pro-tumorigenic processes associated with metabolic dysfunction, including altered energy metabolism, chronic inflammation, and oxidative stress. Current evidence remains largely indirect, and its role in CRC should therefore be interpreted as modulatory rather than definitively protective [265].

Alterations in gamma-glutamyl transferase (GGT), a marker of oxidative stress and hepatic metabolic dysfunction, have been associated with the combined impact of obesity and T2DM on CRC risk in the Saudi population [266].

Beta-hydroxybutyrate (BHB), a key ketone body produced under conditions of reduced glucose availability, has recently been proposed as a potential metabolic modulator with anti-tumorigenic properties. Evidence from in vitro and preclinical models indicates that BHB may inhibit cell proliferation, attenuate inflammation, and regulate gene expression through epigenetic mechanisms, including histone deacetylase (HDAC) inhibition [267]. Although these findings require further validation, they support a potential role for BHB in modulating tumor-promoting metabolic pathways.

Circadian rhythm disruption represents an additional layer linking obesity, metabolic dysfunction, and pathogenesis of CRC. Disruption of the biological clock, driven by irregular feeding patterns, sleep disturbances, and light exposure, can promote insulin resistance, chronic low-grade inflammation, and adipose tissue dysfunction, all of which contribute to tumor development. At the molecular level, circadian dysregulation affects key pathways involved in cell cycle control, DNA repair, and metabolic signaling, thereby influencing both cancer initiation and progression. Circadian rhythms also modulate gut microbiota composition and function, further shaping host metabolism and immune responses. Epidemiological studies consistently report an increased CRC risk in populations exposed to chronic circadian disruption, such as shift workers, suggesting that temporal factors may represent an additional dimension in CRC prevention and therapeutic strategies [268].

Metabolic syndrome and T2DM should therefore be regarded not merely as comorbid conditions, but as systemic drivers of this type of cancer. Through hyperinsulinemia, insulin resistance, hyperglycemia, chronic inflammation, oxidative stress, and broader metabolic dysregulation, these disorders establish a pro-tumorigenic environment that contributes to tumor initiation, shapes disease onset, including at younger ages, and influences progression, prognosis, and therapeutic responses.

### 4.4. Diet–Microbiota Axis and Metabolic Inflammation in CRC

#### 4.4.1. Dietary Patterns, Microbiota, and Metabolic Outputs

Dietary patterns characterized by high consumption of ultra-processed foods (UPFs) are increasingly recognized as key contributors to CRC risk, particularly in the context of obesity. Beyond excess caloric intake, UPFs influence tumorigenesis through combined metabolic, inflammatory, and microbiota-mediated mechanisms. Evidence from a large prospective EPIC cohort study [269] indicates that higher UPF intake is associated with an increased risk of several cancers, including CRC. These associations persist after adjustment for BMI, physical activity, and other lifestyle factors, suggesting that the impact of UPFs is not solely mediated by adiposity and that food processing itself represents an independent dimension of dietary risk.

UPFs often contain additives (e.g. emulsifiers, artificial sweeteners, ASs), neo-formed contaminants generated during high-temperature processing (such as acrylamide), and packaging-related chemicals (including bisphenols), all of which are implicated in oxidative stress, chronic inflammation, and endocrine disruption. These effects converge on pathways central to obesity-driven carcinogenesis, including activation of the insulin/IGF-1 axis and persistent low-grade inflammation.

AS, including aspartame, have not been consistently associated with increased cancer risk in the general population. However, some evidence suggests a potential association in metabolically vulnerable subgroups, such as individuals with T2DM, although it remains unclear whether this reflects direct effects of AS or underlying metabolic alterations, including insulin resistance and chronic inflammation [270].

A dose–response relationship between UPF consumption and cancer risk has been reported and is only partially explained by obesity, indicating the involvement of additional biological mechanisms. UPFs are typically low in dietary fiber and high in refined carbohydrates and unhealthy fats, leading to alterations in gut microbiota composition and reduced production of protective metabolites such as SCFAs, particularly butyrate. Reduced SCFA availability compromises epithelial integrity and anti-inflammatory signaling. Food additives such as emulsifiers can disrupt the mucus layer and increase intestinal permeability, facilitating microbial translocation and activation of pro-inflammatory pathways that contribute to a tumor-promoting microenvironment. In contrast, fiber-rich diets support SCFA production and mucosal homeostasis, whereas Western dietary patterns rich in fat and red or processed meat promote the generation of harmful metabolites, including secondary bile acids, hydrogen sulfide, and N-nitroso compounds, which drive inflammation, DNA damage, and tumorigenesis [271]. The physicochemical properties of dietary fiber, including solubility, fermentability, viscosity, and molecular complexity, critically influence gut microbiota composition and function. By promoting the production of beneficial metabolites such as SCFAs, particularly butyrate, specific fiber types enhance epithelial barrier integrity and modulate inflammatory responses, supporting the potential for personalized dietary interventions [272].

Primary bile acids synthesized in the liver are extensively modified by the gut microbiota into secondary bile acids, which act as signaling molecules through receptors such as farnesoid X receptor and G protein-coupled bile acid receptor (TGR5), regulating lipid and glucose metabolism, intestinal barrier integrity, and immune responses. Dysbiosis disrupts this network, leading to an altered bile acid pool enriched in cytotoxic and pro-inflammatory secondary bile acids. Such alterations have been implicated in the pathogenesis of gastrointestinal disorders, including CRC, as well as systemic metabolic diseases such as obesity and type 2 diabetes. Precision nutritional interventions, such as fiber-rich diets, prebiotics, or targeted modulation of microbial taxa may reshape bile acid profiles and downstream signaling pathways, influencing metabolic and relevant inflammatory processes [273].

A relevant clinical trial is currently evaluating the effects of a high-fiber legume-enriched diet in overweight and obese individuals with a history of noncancerous adenomatous polyps, a population at increased risk for CRC. The intervention assesses its impact on gut microbiota composition and metabolic outputs, including the production of SCFAs such as butyrate, which support epithelial integrity and exert anti-inflammatory and antiproliferative effects. The intervention is expected to accelerate intestinal transit, reducing mucosal exposure to potential carcinogens, while improving systemic parameters linked to obesity-driven carcinogenesis, including insulin sensitivity and chronic low-grade inflammation. Although ongoing, this study may provide important insights into the role of fiber-rich dietary interventions in modulating CRC risk [274].

Dietary patterns enriched in fiber, polyphenols, omega-3 FAs, and selected vitamins may counteract obesity-driven carcinogenesis by modulating key metabolic and inflammatory pathways. Among these, resveratrol exerts beneficial effects on metabolic and inflammatory processes; however, clinical evidence remains heterogeneous and limited by small sample sizes, short intervention durations, and variability in dosing. Its anticancer efficacy in humans therefore remains inconclusive [275].

#### 4.4.2. Microbiota, Host Interactions, and CRC Susceptibility

Microbiota-driven metabolic and immune alterations represent a central interface linking environmental exposures to CRC initiation and progression.

Population-based analyses indicate that obesity-driven tumorigenesis in the colorectum is shaped not only by dietary and metabolic factors but also by host genetic susceptibility. Population-specific genomic alterations and immune signatures interact with the gut microbiome, contributing to distinct tumor-associated microbial profiles enriched in pro-carcinogenic bacteria. These features suggest that CRC disparities arise from the interplay between host genetics, microbiota composition, and inflammatory signaling rather than from socioeconomic factors alone [276].

Metagenomic analyses indicate that individuals with unfavorable lifestyle patterns, characterized by high intake of red and processed meat, low fiber consumption, and excess body weight, exhibit a gut microbiome with reduced diversity and increased abundance of bacteria associated with CRC-promoting processes, including species involved in toxin production, mucin degradation, and pro-inflammatory signaling. In contrast, healthier lifestyle patterns are associated with microbial communities enriched in fiber-fermenting bacteria and metabolic pathways related to SCFA production, particularly butyrate. These shifts are not only compositional but functional, with high-risk profiles linked to increased microbial pathways involved in amino acid fermentation, secondary bile acid metabolism, and the generation of pro-inflammatory and genotoxic metabolites. These alterations contribute to epithelial damage, immune activation, and the establishment of a tumor-promoting microenvironment [277].

Chronic infections further illustrate how microbially driven immune modulation influences tumor initiation and progression in the colorectum. Longitudinal data indicate that intestinal *Clostridioides difficile* infection is associated with an increased subsequent risk of CRC, likely reflecting persistent mucosal inflammation, toxin-mediated epithelial damage, and long-term disruption of microbial metabolic balance [278]. Similarly, epidemiological evidence suggests that *Helicobacter pylori* infection correlates with increased CRC risk, supporting the concept that chronic infection-induced immune activation and systemic inflammatory responses may extend beyond the primary site of colonization [279]. Helminth infections provide an additional model of host–microbiota–immune interaction, as their ability to modulate immune responses and inflammatory tone may influence epithelial homeostasis and cancer susceptibility [280].

Cross-trait genetic association analyses based on genome-wide association study datasets have provided further insight into the shared genetic architecture linking obesity, CRC, and inflammatory bowel disease [281]. Integrative analyses identified pleiotropic loci contributing to the overlap among these conditions, supporting a common biological basis underlying their epidemiological association. Variants mapped to established CRC susceptibility regions, including loci near SMAD7 (18q21), TCF7L2 (10q25), CCND1 (11q13), and the 8q24 region linked to MYC regulation, along with genes involved in immune modulation (e.g., IL23R, Human leukocyte antigen, HLA, *loci*) and metabolic traits (e.g., Fat Mass and Obesity-Associated protein, Melanocortin 4 receptor) [282,283,284]. These shared genetic signals converge on key pathways, including inflammatory signaling, insulin resistance, lipid metabolism, gut barrier function, and host microbiome interactions, collectively promoting colorectal tumorigenesis in genetically susceptible individuals. These findings underscore the importance of integrating genetic susceptibility with metabolic and microbial factors for risk stratification and therapeutic targeting.

## 5. Immune Landscapes and Metabolic Constraints in CRC: Shaping the TME and Response to Immunotherapy

### 5.1. Hot and Cold Tumors: Immune Landscapes of Colorectal Cancer Across Microsatellite Status

CRC exhibits distinct TMEs defined by MSI and MMR status, which shape immune composition and responsiveness to ICIs. MSI-H/dMMR tumors display an immune-inflamed “hot” phenotype, whereas MSS/pMMR tumors are typically immune-suppressed or immune-excluded “cold” tumors [285,286].

MSI-H/dMMR CRC arises from defective DNA MMR, leading to high TMB and neoantigen generation that drives CD8^+^ T-cell activation [287]. These tumors are characterized by dense infiltration of tumor-infiltrating lymphocytes (TILs), particularly cytotoxic CD8^+^ T cells in the tumor core and invasive margins, with high expression of granzyme A, granzyme B, perforin, and cytokines such as Interferon gamma (IFN-γ) and TNF-α [288,289]. Notably, CD8^+^ T-cell infiltration correlates with improved survival independently of MMR status, *Polymerase ε* (*POLE*) mutation, or chromosomal instability [289,290]. Persistent antigen exposure in MSI-H tumors induces adaptive immune resistance through upregulation of inhibitory checkpoints such as PD-1, CTLA-4, and Lymphocyte Activation Gene 3 on T cells and PD-L1 on tumor and antigen-presenting cells. Nevertheless, a subset of progenitor-like exhausted CD8^+^ T cells retains proliferative potential and can be reinvigorated by checkpoint blockade, supporting the clinical efficacy of ICIs in this subtype [291].

Beyond CD8^+^ T cells, MSI-H CRCs are enriched in T helper (Th)1-polarized CD4^+^ T cells, natural killer (NK) cells, and functionally active DCs, which often localize at invasive margins and contribute to tertiary lymphoid structure (TLS) formation, sustaining antigen presentation and local immune priming [288]. Immunosuppressive populations, including MDSCs and M2-like TAMs, are comparatively limited [289]. A prominent feature of MSI-H CRC is the enrichment of B cells, particularly proliferating Ki67^+^ CD79a^+^ cells, IgG^+^ memory B cells, and plasma cells expressing CD27, CD38, and CD138, organized within TLS-like structures at invasive margins, supporting antigen presentation, Ab production, and coordination of T-cell responses. Accordingly, TLS presence correlates with improved prognosis and responsiveness to ICIs [292,293,294]. Nevertheless, tumor-intrinsic alterations can undermine this immunogenicity: defects in antigen presentation (e.g. β2-microglolin or HLA loss) impair T-cell recognition [295] and disruption of IFN-γ signaling (e.g. *JAK1/2* mutations) promotes immune escape [296]. Moreover, oncogenic pathways such as Wnt/β-catenin, MAPK, and PI3K limit DC recruitment, alter chemokine production, and promote immunosuppressive myeloid populations, contributing to T-cell exclusion or dysfunction [297,298]. In parallel, TGF-β-driven stromal remodeling can physically exclude T cells. At the same time, chronic IFN-γ exposure sustains checkpoint molecule expression, resulting in a “hot but exhausted” phenotype that is yet therapeutically targetable [299,300]. In contrast, MSS/pMMR CRC is largely defined by low immunogenicity and resistance to immunotherapy, a concept further refined by the consensus molecular subtype (CMS) classification, which integrates tumor-intrinsic and immune features into 4 groups [301,302].

CMS1 (“immune subtype”) overlaps with MSI-H tumors and is characterized by high TMB, strong neoantigen-driven immune activation, and infiltration by Th1, CD8^+^ T cells, NK cells, and DCs. In contrast, CMS2 (“canonical”) and CMS3 (“metabolic”) tumors are typically MSS, poorly infiltrated, and display immune-desert phenotypes with limited DC presence and weak T-cell activation. CMS3 additionally exhibits metabolic rewiring and frequent KRAS mutations. CMS4 (“mesenchymal”) tumors, although often infiltrated, are dominated by stromal activation, TGF-β signaling, and immunosuppressive populations such as Tregs and MDSCs, resulting in poor prognosis. Notably, many CRCs do not fit neatly into a single CMS category, reflecting substantial biological heterogeneity. Mechanistically, MSS/pMMR tumors exhibit low TMB and limited neoantigen availability, resulting in insufficient priming of tumor-specific T cells. CD8^+^ T cells are sparse, often confined to the tumor periphery, and functionally impaired by checkpoint signaling, metabolic stress, suppressive cytokines, and inhibitory myeloid interactions [289]. Single-cell analyses reveal that most infiltrating CD8^+^ T cells are bystander rather than tumor-specific clones, with limited clonal expansion and reduced exhaustion marker expression associated with effective reinvigoration [291,303].

The CD4^+^ T-cell compartment is skewed toward immunosuppressive phenotypes, with Tregs and Th17 cells promoting immune evasion via IL-10 and TGF-β secretion and recruitment of suppressive myeloid cells. IL-17A further drives tumor progression and angiogenesis through IL-6/STAT3 signaling, whereas IL-17F may exert anti-tumoral effects; however, the overall cytokine milieu remains tolerogenic [289,304,305,306]. Additional CD4^+^ subsets, including Th2, Th22, and T follicular helper (Tfh) cells, play context-dependent roles, with Tfh cells supporting beneficial B-cell responses, while IL-22 has been linked to chemoresistance [307].

The γδ T cell role appears to evolve dynamically during tumor development, contributing to early tumor surveillance independently of MHC, and exerting either anti-tumor (IFN-γ producing) or pro-tumor (IL-17 producing) effects. Notably, these cells may mediate responses to immunotherapy even in MHC class I-deficient dMMR CRC [308,309], challenging the paradigm that only highly mutated tumors respond to ICB. Tumor-intrinsic pathways further reinforce immune exclusion in MSS CRC, particularly the WNT/β-catenin signaling, which impairs the recruitment of CD103^+^ DCs and subsequent CD8^+^ T-cell priming [310,311]. TGF-β signaling also plays a critical role, promoting stromal fibrosis, extracellular matrix deposition, and direct suppression of cytotoxic T-cell differentiation while stabilizing Tregs and polarizing macrophages toward an M2-like phenotype [312].

**Figure 3:**
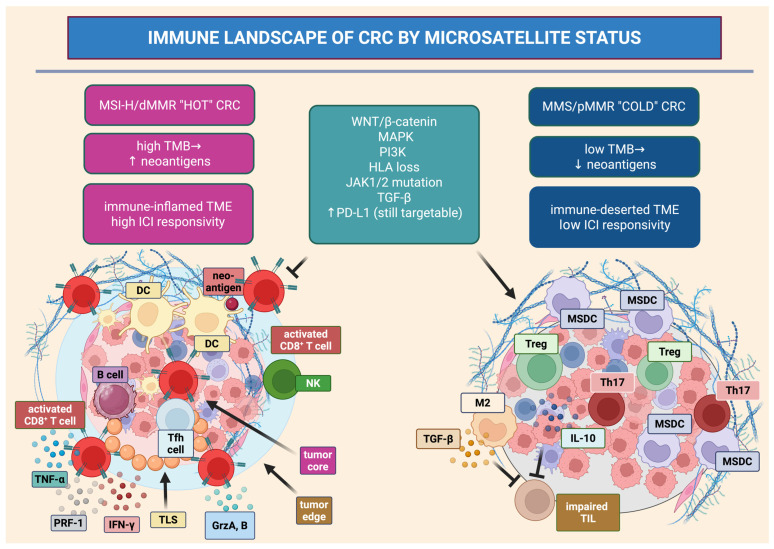
Immune landscape of CRC according to microsatellite status. Comparison of the immune composition and immunological features of MSI-H/dMMR (“hot”) and MSS/pMMR (“cold”) CRC. MSI-H/dMMR tumors are characterized by increased neoantigen load, immune-cell infiltration, and responsiveness to ICIs, whereas MSS/pMMR tumors display immune exclusion or immune suppression associated with reduced responsiveness to immunotherapy. Major molecular mechanisms contributing to immune evasion are also shown. Created with BioRender (https://app.biorender.com). Abbreviations: CD8, cluster of differentiation 8; CRC, colorectal cancer; DC, dendritic cell; dMMR, deficient mismatch repair; GrzA/B, granzyme A/B; HLA, human leukocyte antigen; ICI, immune checkpoint inhibitor; IFN-γ, interferon gamma; IL-10, interleukin 10; JAK1/2, Janus kinase 1/2; MAPK, mitogen-activated protein kinase; MDSC, myeloid-derived suppressor cell; MSI-H, microsatellite instability-high; MSS, microsatellite stable; NK, natural killer; PD-L1, programmed death ligand 1; PI3K, phosphoinositide 3-kinase; pMMR, proficient mismatch repair; PRF-1, perforin 1; TGF-β, transforming growth factor beta; Th17, T helper 17 cell; Tfh, T follicular helper cell; TIL, tumor-infiltrating lymphocyte; TLS, tertiary lymphoid structure; TMB, tumor mutational burden; TME, tumor microenvironment; TNF-α, tumor necrosis factor alpha; Treg, regulatory T cell; WNT, Wingless/Integrated.

The innate immune compartment amplifies this suppression, with MDSCs inhibiting T and NK cells via arginase-1 activity and nitric oxide (NO) production, TAMs adopting predominantly M2-like pro-tumorigenic phenotypes, and DCs remaining scarce and functionally impaired due to VEGF, prostaglandin E_2_, and TGF-β [289]. B-cell responses are also diminished, with reduced proliferation, plasma cell differentiation, and TLS formation, reflecting impaired humoral immunity [292,293]. Collectively, MSS/pMMR CRC represents an immune-excluded, low-antigenicity state driven by oncogenic signaling, stromal barriers, and multilayered immunosuppressive networks, in contrast to the inflamed but checkpoint-restrained MSI-H/dMMR phenotype. Intermediate tumor states also exist, characterized by either immune infiltration with suppression or T-cell exclusion at the invasive margin, further underscoring the complexity of CRC immune landscapes and the need for rational combination strategies to overcome primary resistance to immunotherapy. The main immunological features of MSS and MMR CRC are depicted in Figure 3.

### 5.2. Metabolic Reprogramming of Immune Cells Within the TME of CRC and Immunological Determinants of Response to Icis

During tumorigenesis, cancer cells undergo metabolic rewiring to sustain proliferation and adapt to the complex, often hostile TME [313,314]. Because tumor and immune cells coexist within a confined space, they compete for essential nutrients, including glucose, amino acids, lipids, and oxygen, required for both tumor growth and immune effector function. In CRC, this competition is particularly pronounced, generating a nutrient-depleted, hypoxic, and acidic TME. Tumor cells display high glucose uptake through aerobic glycolysis, driven by GLUT1 overexpression and oncogenic signaling, creating steep intratumoral glucose gradients associated with advanced stage, lymph node metastasis, and reduced OS [31,32,33,34,35]. This metabolic pressure severely restricts immune cell fitness, as effector CD8^+^ T cells and NK cells depend on glycolysis for proliferation, cytokine production (e.g., IFN-γ), and cytotoxicity.

At the tumor level, CRC cells display enhanced glycolysis (PKM2, HK2, GLUT1), increased glutamine uptake (ASCT2/*SLC1A5*), and active one-carbon metabolism, all associated with proliferation, metastasis, and poor prognosis [31,315,316,317,318]. These metabolic programs are shaped by genetic and epigenetic alterations, including APC/β-catenin activation, *TP53* loss, and *KRAS* mutations, which collectively reinforce metabolic addiction and immune evasion [319]. A hallmark consequence is a persistent lactate accumulation and extracellular acidification, which broadly suppress anti-tumor immunity [320].

Recent reviews have provided a comprehensive overview of how metabolic reprogramming within the TME influences CRC progression, immune evasion, and responsiveness to immunotherapy [321,322]. Beyond glucose restriction, CRC tumors deplete amino acids and lipids while generating immunosuppressive metabolites. Activated T cells require ASCT2-mediated glutamine uptake to sustain proliferation and cytokine production (IL-2/IFN-γ) [323]. Glutamine depletion impairs CD8^+^ T cell and NK cell proliferation while promoting Treg differentiation [324]. Arginine depletion, driven by tumor uptake [325] and arginase activity in myeloid cells [326], impairs TCR signaling, cytokine production, and immune synapse formation [322]. Similarly, tryptophan depletion and kynurenine accumulation via IDO/TDO pathways induce T-cell exhaustion and apoptosis through Aryl hydrocarbon receptor signaling and promote immunosuppressive cell expansion [327]. Additional amino acid deprivation, including methionine, alanine, and cystine limitation [328,329,330], further induces metabolic stress and epigenetic reprogramming in immune cells [331].

A defining feature of CRC metabolism is lactate accumulation, which acidifies the TME and acts as both a metabolic byproduct and signaling molecule. Lactate suppresses Nuclear factor of activated T cells signaling and IFN-γ production in CD8^+^ T cells, while promoting Treg differentiation and PD-1 expression [332]. It also induces NK cell dysfunction and immune escape mechanisms [333,334]. Mechanistically, lactate promotes immune suppression via G protein-coupled receptor 81 signaling, PD-L1 induction, and antigen presentation impairment [335,336,337]. Emerging evidence further highlights histone lactylation as an epigenetic mechanism linking metabolic configuration to immune suppression [338]. Hypoxia reinforces these effects via HIF-1α, which integrates oncogenic signaling (PI3K/AKT/mTOR, MAPK) to regulate glycolysis, angiogenesis, and pH control, thereby amplifying immune suppression [339,340,341].

Lipid metabolic reprogramming is another key feature of CRC. Increased FA synthesis and uptake create a lipid-rich environment that supports Treg and TAM survival via FAO. Lipid overload induces CD8^+^ T cell dysfunction, ER stress, and exhaustion, while cholesterol metabolites enhance immune checkpoint expression [342,343]. Prostaglandin E2 and other lipid mediators further suppress cytotoxic immunity and promote the expansion of immunosuppressive cells [344]. Immune subsets display distinct metabolic programs. Effector T cells rely primarily on glycolysis, whereas Tregs depend on FAO and OXPHOS, allowing persistence in nutrient-deprived environments [345,346,347]. Lactate exposure further enforces this dichotomy by suppressing effector T-cell function and favoring Treg survival [348,349].

Myeloid cell populations are likewise reprogrammed to support immune escape. TAMs adopt an M2-like phenotype driven by lactate, lipid availability, and cytokines such as IL-4 and TGF-β, relying on FAO and OXPHOS for their function [337,344,350]. MDSCs further exploit FAO, glutaminolysis, and HIF-1α signaling to suppress T-cell and NK-cell activity through arginase-1, NO, reactive oxygen species (ROS), and PD-L1 upregulation [351,352,353,354,355,356]. DCs are similarly metabolically constrained, leading to impaired antigen presentation and a tolerogenic phenotype under lipid and glucose stress [342,343,357]. In addition, B-cell responses are markedly reduced in MSS CRC, particularly at invasive margins. These tumors exhibit limited B-cell proliferation, reduced plasma cell differentiation, and poor formation of TLS, reflecting an impaired humoral immune response. Spatial analyses confirm the absence of organized immune niches typical of immunologically active tumors [292,293].

Overall, these mechanisms establish an immunometabolic network in CRC in which immune cells are nutrient-deprived, exposed to inhibitory metabolites, and reprogrammed into dysfunctional states, promoting immune escape and therapy resistance [321].

### 5.3. Immunological Determinants of Response to ICIs

Response to ICIs in CRC is largely driven by tumor immunogenicity, particularly the presence of dMMR or MSI-H status. Hallmarks of responsiveness include high TMB, abundant CD8^+^ TILs, PD-L1 expression, and a “hot” TME. Conversely, immunosuppressive components, such as Tregs, MDSCs, and specific gut microbiome profiles, can impair therapeutic efficacy. As recently reported [358], numerous biomarkers have been investigated to predict response to ICB in CRC, including TME characteristics, genomic alterations, CMS, and microbiota composition. While the clinical benefit of ICB in dMMR/MSI CRC is well established in prospective trials [359,360,361], identifying reliable predictive markers in proficient MMR (pMMR)/MSS) CRC, which represents the majority of cases, remains a major unmet need.

TILs are widely recognized as predictors of ICB response across several tumor types, although their role in CRC is less consistent [362]. In CRC, high densities of CD3^+^, CD4^+^, CD8^+^, and CD20^+^ TILs generally reflect effective antitumor immunity, whereas Forkhead box P3 (FOXP3)^+^ Tregs are associated with immunosuppression and poorer outcomes [363,364]. Moreover, not only the density but also the spatial distribution of TILs, particularly CD3^+^, CD8^+^, and FOXP3^+^ cells within the tumor core and invasive margin, correlates with disease-free and OS [365], consistent with meta-analytic data [366]. High immune infiltration has been linked to increased PD-L1 expression and favorable prognosis [367] and, in some contexts, Treg infiltration has paradoxically correlated with improved outcomes in chemo-immunotherapy settings [368]. Conversely, post-hoc analyses of the atezoTRIBE study suggest that higher TIL levels may be associated with reduced benefit from atezolizumab in metastatic CRC [369]. This variability likely reflects substantial heterogeneity in TIL composition, indicating that quantitative assessment alone, without functional characterization, may be insufficient, particularly in pMMR/MSS CRC [369].

The Immunoscore, based on CD3^+^ and CD8^+^ T-cell densities in the tumor core and invasive margin, is a validated prognostic tool in stage I-III CRC [370] that generates a score from 0 to 4 predicting recurrence risk and supporting clinical decision-making in resected disease [370]. Its predictive value for ICB response is still being defined; however, higher Immunoscore values or T-cell densities have been associated with improved outcomes in dMMR CRC treated with pembrolizumab and in neoadjuvant ICI trials (e.g., NICHE and NICHE-2), where most dMMR tumors, and a subset of pMMR tumors, achieved major pathological responses [371]. These results suggest a potential role for Immunoscore in patient selection. However, limitations include interobserver variability in immunohistochemistry (IHC), the need for adequate sampling, and intratumoral heterogeneity [370]. Importantly, ICI efficacy depends on the broader immune context, including Tregs, NK cells, DCs, and B cells, rather than CD8^+^ T cells alone [370].

PD-L1 expression, routinely used as a predictive biomarker in other malignancies, has a more limited and controversial role in CRC [372]. Approximately half of CRCs show PD-L1 expression at a 1% cutoff [373], particularly in metastatic lesions and in association with MSI, *BRAF* mutations, medullary histology, and cytotoxic TILs [374]. However, its clinical utility is limited by spatial heterogeneity [375], dynamic regulation during disease progression [376], and lack of assay standardization [377]. Notably, pembrolizumab demonstrates durable efficacy in dMMR/MSI CRC regardless of PD-L1 status [360]. Combined assessment of PD-L1 and TILs may improve predictive accuracy, although prospective validation is still needed [378,379]. Overall, MSI-H and dMMR remain the most robust predictive biomarkers for ICB response in CRC, reflecting high neoantigen load and immune activation [380]. Their predictive value has been consistently demonstrated in both metastatic and localized settings treated with immunotherapy [188,189,360,361,381,382]. In clinical practice, MMR IHC is more widely used than next-generation sequencing-based MSI testing due to accessibility. Overall, current evidence suggests that no single biomarker is sufficient to predict ICI responsiveness across all CRC subtypes, supporting the development of integrated models that combine genomic, immune, and immunometabolic features.

TMB has shown predictive value in melanoma and non-small-cell lung cancer. Still, its role in CRC is less clear [383]. Although a subset of pMMR/MSS tumors with high TMB may respond to ICB [384], other studies report limited benefit, reducing its value as a standalone biomarker [385,386]. *POLE* mutations, present in approximately 1% of CRCs and typically mutually exclusive with MSI [387], generate hypermutated tumors with strong immunogenicity [388,389]. These tumors are associated with favorable prognosis, increased checkpoint expression, and improved responses to ICB [359,390,391]. However, response may vary depending on the specific mutation [392].

*BRAF* V600E mutations, typically associated with right-sided tumors and poor prognosis [393], do not appear to negatively affect ICB responsiveness in MSI-H/dMMR CRC. Subgroup analyses from KEYNOTE-177 and CheckMate 142 showed comparable outcomes between *BRAF*-mutant and wild-type tumors [360,361]. Although not a direct predictive biomarker [394], its association with inflammatory phenotypes suggests indirect relevance. In contrast, activation of the WNT/β-catenin pathway is associated with immune exclusion and reduced ICB efficacy [395].

As reported above, CMS integrates genomic, transcriptomic, and immune features into four groups: CMS1 (immune), CMS2 (canonical), CMS3 (metabolic), and CMS4 (mesenchymal) [301,396]. CMS1 tumors are enriched for MSI, immune infiltration, and inflammatory signaling, making them the most responsive to ICB [397]. CMS4 tumors, despite immune infiltration, are characterized by stromal activation and immunosuppression, suggesting a need for combination strategies rather than ICB monotherapy, whereas CMS2 and CMS3 are generally immune “cold.” Notably, CMS1 encompasses a broader subset of metastatic CRC than MSI/dMMR alone [398].

Emerging transcriptomic data further refine this framework, showing that stromal-high and proliferation-low signatures are associated with poorer ICB outcomes even in MSI-H CRC [399]. Overall, CMS classification provides a promising multidimensional approach to patient stratification, although standardization and prospective validation remain necessary [358,396].

### 5.4. Dietary Modulation of Antitumor Immunity: The Diet/Immune/Microbiota Axis and Its Impact on Intratumoral Immunity and Response to ICIs in CRC

CRC development reflects a complex interplay among diet, vitamin D status, BMI, microbiota composition, inflammation, and adipokine signaling, although these relationships remain only partially understood [400]. Among these factors, diet plays a central role by shaping gut microbiota composition and, consequently, modulating tumor immunity and responses to immunotherapy.

Dietary patterns exert distinct immunological effects. Fiber-rich diets, such as the Mediterranean diet, promote beneficial microbial taxa, including Bacteroidetes and SCFA-producing bacteria. In contrast, Western diets, characterized by high fat and low fiber intake, induce dysbiosis, chronic inflammation, and immune dysfunction [401]. A key mechanistic link between diet and immunity is the production of microbial metabolites. SCFAs modulate immune responses by promoting Treg expansion, enhancing CD8^+^ T-cell activation, and reinforcing epithelial barrier integrity, thereby limiting systemic inflammation [402]. However, SCFAs exert context-dependent effects in CRC. While butyrate is widely recognized for its anti-inflammatory and tumor-suppressive properties, acting as a HDAC inhibitor and promoting regulatory immune responses that help counteract the inflammation driving CRC development [267], it can, under certain conditions, also exert pro-inflammatory or even tumor-promoting effects. These opposing outcomes may arise in the presence of altered metabolic states or changes in receptor expression. SCFAs exert their effects primarily through G protein coupled receptors, such as Free Fatty Acid Receptor 2/3 and Hydroxycarboxylic acid receptor 2, with downstream responses shaped by cell type, receptor availability, and the tumor context. In addition, dysbiotic conditions characterized by excessive or imbalanced metabolite production may further shift their role, amplifying inflammation rather than resolving it [403]. Notably, in preclinical models, butyrate enhances anti-tumor immunity by promoting both the generation and function of effector CD8^+^ T cells in vitro [404].

Additional microbial metabolites, such as tryptophan derivatives, further influence immune-cell epigenetic programming and cytokine signaling, contributing to a more immunogenic TME [405]. Conversely, dysbiosis supports tumor-supportive immune environments. Pathobionts such as *Fusobacterium nucleatum* are associated with reduced CD3^+^/CD8^+^ T-cell infiltration, increased recruitment of immunosuppressive myeloid cells, and elevated pro-inflammatory cytokines (IL-1β, IL-6, TNF-α), collectively facilitating immune evasion [401,406]. These effects are further amplified by the recruitment of MDSCs and M2-like macrophages, along with activation of TLR-NF-κB signaling pathways, which sustain chronic inflammation and immune escape [403].

Diet-induced alterations in the microbiota also shape innate immunity. Balanced microbial ecosystems limit MDSC expansion and M2 macrophage polarization, whereas high-fat diets promote M2 polarization and tumor progression [407]. At the interface of innate and adaptive immunity, beneficial microbiota enhances DC maturation and antigen presentation, thereby improving T-cell priming and increasing CD8^+^ T-cell infiltration [406]. Consistently, responders to ICB often exhibit greater microbiome diversity, enriched in SCFA-producing and immunostimulatory taxa such as *Bifidobacterium* and *Clostridiales* [402]. In particular, *Akkermansia muciniphila* has been linked to increased IL-12 production and enhanced recruitment and priming of cytotoxic T cells [408]. Importantly, microbiota-driven immune modulation extends beyond metabolite production and includes direct microbe–host interactions, such as adhesion molecules and signaling pathways that influence epithelial integrity and immune activation [403].

These findings support dietary interventions to improve immunotherapy efficacy. High-fiber diets are associated with increased T-cell activation and interferon-related gene signatures during ICB, while ketogenic diets show preclinical potential in enhancing effector T-cell responses and reducing PD-L1-mediated immunosuppression [409]. Furthermore, fecal microbiota transplantation from responders can restore sensitivity to ICIs in preclinical and early clinical settings, supporting a causal role of the microbiome [406]. Nevertheless, these effects are highly context-dependent. Variability in baseline microbiota composition, host metabolism, and dietary patterns likely explains inconsistent findings across studies, including reports showing no uniform benefit of dietary fiber on ICB efficacy [410].

Clinically, although microbiome diversity and specific bacterial signatures are associated with improved immunotherapy responses across multiple cancers, CRC remains relatively less responsive, except in MSI-H or dMMR subgroups [411]. Consequently, current research is focusing on dietary modulation strategies, including prebiotics, high-fiber interventions, and defined dietary patterns, to enhance ICB efficacy through microbiome remodeling [409]. Precision nutrition approaches integrating microbiome and immune profiling may further enable personalized interventions [412].

Targeted nutritional interventions may further potentiate immunotherapy. Methionine restriction, for instance, increases PD-L1 expression via IFN-γ signaling and modulates MHC-I expression through cGAS-STING pathways, ultimately enhancing ICI efficacy in preclinical CRC models, with sex- and age-dependent effects [413]. Similar immunomodulatory effects have been observed with caloric restriction mimetics such as metformin and resveratrol. In preclinical CRC models, caloric restriction and related metabolic interventions slow tumor growth and reshape the TME through immune-mediated mechanisms. These strategies reduce systemic glucose levels and insulin/IGF-1 signaling, leading to decreased tumor proliferation and a more immunostimulatory environment. Such changes are associated with increased CD8^+^ T-cell infiltration and reduced immunosuppressive populations, including MDSCs and Tregs, supporting an immune-mediated component of tumor control [402].

Metabolic rewiring of T cells has been observed in CRC, often in the context of dietary interventions rather than classical dietary restriction alone. For example, ketogenic diets or fasting-mimicking interventions increase circulating ketone bodies, which serve as alternative energy sources for T cells. This enhances mitochondrial function, OXPHOS, and effector differentiation, ultimately improving CD8^+^ T-cell activity and reducing exhaustion phenotypes [409]. Overall, methionine restriction appears to enhance anti-tumor immune responses and improve immunotherapy efficacy, with potential sex-specific differences [413]. In parallel, diet-induced metabolic changes influence macrophage polarization: high-fat diets promote M2-like macrophage phenotypes and tumor progression, whereas metabolic interventions can reverse this effect and restore antitumor immunity, indirectly supporting CD8^+^ T-cell function [407].

Although direct evidence of ketolysis-dependent CD8^+^ T-cell reprogramming under dietary restriction in CRC remains limited, multiple studies support a broader framework in which metabolic interventions enhance mitochondrial fitness, promote effector differentiation, reduce T-cell exhaustion, and synergize with ICIs, particularly anti-PD-1/PD-L1 therapies. This is further supported by evidence that microbiota-derived metabolites, including SCFAs, and diet-induced metabolic shifts enhance CD8^+^ T-cell activation and persistence in CRC [401]. The field is increasingly moving toward integrating dietary restriction, ketogenic strategies, and targeted metabolic interventions as adjuvants to immunotherapy. However, more CRC-specific mechanistic studies are needed to fully validate these approaches. Notably, such interventions may shift macrophage polarization from pro-tumor M2-like states to antitumor phenotypes, thereby indirectly enhancing CD8^+^ T-cell function and counteracting high-fat diet-driven tumor progression [407]. From a translational perspective, microbiome-based biomarkers show promising diagnostic and predictive potential. While traditional CRC screening models achieve accuracies of 66–85% [414], combined microbiome–lifestyle models can exceed 90% accuracy [415]. However, current evidence is limited by small cohort sizes, a lack of external validation, reliance on single-time-point sampling, and biases inherent to dietary assessment tools [416,417].

In summary, diet, microbiota, and immunity form an interconnected axis that influences CRC risk and response to immunotherapy. Although CRC-specific randomized evidence remains limited, multiple strategies, including high-fiber diets, ketogenic interventions, caloric and methionine restriction, and microbiome-targeted therapies, are actively under investigation. Collectively, these approaches aim to enhance ICB efficacy by coordinating metabolic and microbial reprogramming, paving the way for precision nutrition in cancer care.

## 6. An Integrative Immunometabolic Model of CRC: From Risk to Precision Therapy

In this review, we propose a comprehensive and integrative framework for CRC, conceptualizing the disease as a dynamic network of reciprocal interactions between tumor and immune compartments, embedded within systemic and environmental layers. Specifically, tumor-intrinsic programs, systemic alterations, adipose tissue biology, and diet–microbiota interactions are integrated within a shared immunometabolic continuum.

These layers do not operate in isolation but are functionally interdependent, collectively shaping both cancer cell behavior and immune cell function. Tumor-driven rewiring, through nutrient competition, metabolite accumulation, and microenvironmental conditioning, actively suppresses or reprograms immune cell metabolism. In turn, immune cells adapt their functional states in ways that can either restrain or promote tumor progression.

This coordinated network ultimately determines immune contexture and contributes to the establishment and maintenance of immunologically “cold” tumors. Within this perspective, CRC can be viewed as a dynamically evolving immunometabolic ecosystem in which tumor metabolic activity, host systemic conditions, adipose signaling, microbiota-derived cues, and the TME co-evolve rather than function as isolated drivers [322,418].

At the tumor level, coordinated rewiring across CMSs, including glycolysis, glutamine and one-carbon metabolism, lipid synthesis, FAO, and autophagy, establishes a nutrient-depleted yet metabolite-rich TME. This environment suppresses CD8^+^ T cells and NK cells while promoting Tregs, MDSCs, and M2-like macrophages. In parallel, mitochondrial OXPHOS programs and FASN-dependent ferroptosis resistance define “high-power” tumor states associated with immune exclusion and therapeutic resistance [83,419,420].

These effects are subtype-specific: MSI-H tumors retain immune infiltration but exhibit constrained effector function, whereas MSS tumors display coordinated suppression coupled with immune exclusion. This tumor–immune interface reveals multiple actionable vulnerabilities. Inhibition of Wnt/β-catenin or TGF-β signaling restores DC recruitment [311,312], while targeting immunometabolic checkpoints such as arginase, IDO1, and related pathways alleviates immunosuppression [421,422]. These approaches support combinatorial strategies aimed at enhancing tumor antigenicity while relieving constraints within the TME. Cytotoxic chemotherapy induces immunogenic cell death (ICD) and promotes T-cell priming [423], whereas pharmacological xenogenization using alkylating agents such as dacarbazine and temozolomide increases TMB [424,425] and sensitizes CRC to ICIs, as demonstrated in the ARETHUSA trial [426].

Targeted interventions further potentiate these effects. Inhibition of LDHA or IDO1 restores T-cell effector function [422], modulation of the kynurenine pathway (e.g., via caffeine) enhances antitumor immunity [427], and targeting serine biosynthesis or HIF-1α signaling integrates hypoxia, redox homeostasis, and immune regulation [428]. Lipid metabolism emerges as a central hub linking tumor progression, immune suppression, and systemic conditions. Lipid accumulation promotes immunosuppressive phenotypes, whereas inhibition of FAO or FASN enhances chemosensitivity and responsiveness to ICIs [429,430,431,432].

Adipose tissue extends this axis at the systemic level. Adipocyte-derived FAs fuel tumor FAO via CD36, supporting metabolic flexibility, particularly in KRAS-mutant CRC through CPT1A-dependent OXPHOS [430,433]. At the same time, this lipid-rich environment sustains FAO-dependent Tregs and MDSCs and contributes to CD8^+^ T-cell dysfunction [434]. Obesity amplifies these effects through IL-6, TNF-α, and leptin signaling, activating STAT3/NF-κB pathways that promote immune evasion, mesenchymal CMS4 phenotypes, and resistance to immunotherapy [301,434,435]. Its impact is context-dependent: obesity disrupts TLSs in MSI-H tumors, while reinforcing immune exclusion and Th17/IL-17-driven progression in MSS disease [436,437]. Notably, dietary interventions can partially restore CD8^+^ T-cell function and improve immunotherapy efficacy [438].

The gut microbiota provides an additional integrative layer linking diet, host metabolism, and immunity. Dysbiosis alters key metabolites such as short-chain FAs, bile acids, and indoles, which regulate both immune responses and cellular programs, while pathobionts such as *Fusobacterium nucleatum* promote tumorigenesis and immune evasion [322,403,418,439].

**Figure 4:**
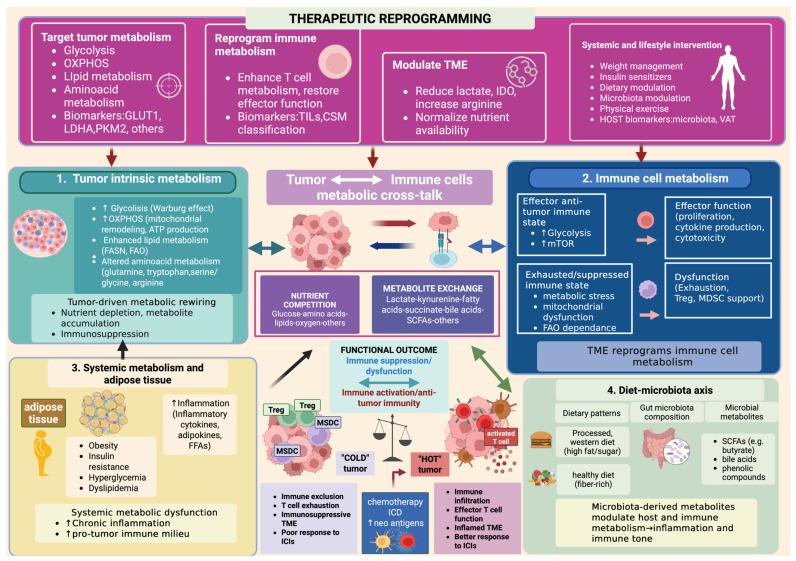
Integrated immunometabolic framework of colorectal cancer (CRC). Schematic overview of the bidirectional interactions among tumor-intrinsic metabolism, immune-cell metabolism, systemic metabolic dysfunction, and the diet–microbiota axis, highlighting potential therapeutic reprogramming strategies targeting metabolic and immune determinants of tumor progression and treatment response. Created with BioRender. Abbreviations: CRC, colorectal cancer; CSM, consensus molecular subtype; FAO, fatty acid oxidation; FFAs, free fatty acids; GLUT1, glucose transporter 1; HK2, hexokinase 2; ICD, immunogenic cell death; IDO, indoleamine 2,3-dioxygenase; LDHA, lactate dehydrogenase A; MDSC, myeloid-derived suppressor cell; mTOR, mechanistic target of rapamycin; OXPHOS, oxidative phosphorylation; PKM2, pyruvate kinase M2; SCFAs, short-chain fatty acids; TIL, tumor-infiltrating lymphocyte; TME, tumor microenvironment; Treg, regulatory T cell; VAT, visceral adipose tissue.

Together, these dimensions converge into an “immunometabolic–adipose therapeutic triangle,” in which tumor biology, immune contexture, and systemic factors are co-targeted through multimodal strategies, including chemotherapy, metabolic inhibitors, microbiota modulation, epigenetic therapies, and lifestyle interventions [403,440,441,442,443]. Within this framework, the therapeutic landscape of CRC is defined by the need to address immunometabolic complexity through rational combinations. Cytotoxic regimens (FOLFOX, FOLFIRI, CAPEOX) remain foundational, not only for their cytotoxic effects but also for their ability to induce ICD, enhance antigen presentation, and reshape systemic inflammation. ICIs are highly effective in MSI-H CRC; however, their limited efficacy in MSS tumors underscores the necessity of overcoming metabolic suppression and immune exclusion. Accordingly, emerging strategies aim to convert “cold” tumors into immunologically responsive states through coordinated immunometabolic adaptation. This perspective supports a shift toward integrated stratification combining tumor markers (GLUT1, PKM2, LDHA), immune parameters (Immunoscore, CMS, TILs), and host-related features (visceral adiposity, metabolic comorbidities, microbiota composition), alongside longitudinal monitoring and computational modeling approaches. Figure 4 highlights key actionable nodes linking metabolic rewiring to immune dysfunction, providing a rationale for combinatorial therapeutic strategies.

## 7. Conclusions

This review supports a conceptual shift, positioning CRC as a dynamic immunometabolic ecosystem in which tumor progression and therapeutic response emerge from continuous interactions among cellular metabolism, immune function, and systemic metabolic context. CRC emerges as a dynamic immunometabolic system in which metabolic constraints shape immune responsiveness.

This perspective implies that successful clinical translation will require moving beyond single-pathway targeting toward adaptive, multi-layered interventions stratified according to metabolic–immune phenotypes rather than purely genetic alterations. It further suggests that future CRC management will increasingly rely on real-time monitoring of immunometabolic states, enabling dynamic treatment adaptation and early identification of resistance trajectories. In this context, metabolic alterations, whether at the level of enzymes, transporters, signaling kinases, transcriptomic signatures, or circulating hormones, emerge as increasingly relevant biomarkers that capture functional tumor–host interactions beyond static molecular classifications. Importantly, glycolytic, mitochondrial, lipid, amino acid, and autophagy-related programs should not be viewed as independent biomarkers. Rather, their coordinated activation generates distinct immunometabolic states that determine tumor fitness, immune exclusion, and therapeutic vulnerability. Accordingly, integrating these metabolic features with established molecular classifiers and clinical parameters may substantially refine patient stratification and uncover context-specific therapeutic vulnerabilities, thereby enabling more precise and mechanistically informed treatment selection.

Ultimately, this framework positions immunometabolic reprogramming as a unifying principle for precision oncology, with the potential to transform CRC from a largely refractory disease in advanced stages into a condition amenable to durable immune-mediated control. In this context, therapeutic success depends on coordinated, patient-specific reprogramming of metabolism, immunity, and systemic influences, enabling the rational conversion of resistant “cold” tumors into treatment-responsive states and advancing precision oncology toward a truly predictive and actionable paradigm.

## Figures and Tables

**Table 2:** Immunometabolic configurations in colorectal cancer and their potential therapeutic implications.

Immunometabolic Configuration	Dominant Features	Resistance Pattern	Potential Therapeutic Opportunities
Glycolytic/proliferative	*GLUT1*, *HK2*, *PKM2*, *LDHA*	Chemotherapy resistance, immune suppression	Glycolysis inhibitors, LDH inhibitors, chemotherapy combinations
Stress-adaptive	*AMPK*, *PDK4*, glutamine metabolism, autophagy	Survival under metabolic stress, chemoresistance	Autophagy inhibitors, glutamine-targeting strategies
Lipid/FAO-immunosuppressive	*CPT1A*, *FAO*, *FASN*, *IDO1*	ICI resistance	FAO inhibition, FASN targeting, immunotherapy combinations
OXPHOS-dependent	*PGC-1α*, *NDUFA4L2* OXPHOS signatures	Chemotherapy resistance	Mitochondrial/OXPHOS-targeted therapies

Abbreviations: AMPK, AMP-activated protein kinase; CPT1A, carnitine palmitoyltransferase 1A; FAO, fatty acid oxidation; FASN, fatty acid synthase; GLUT1, glucose transporter 1; HK2, hexokinase 2; ICI, immune checkpoint inhibitor; IDO1, indoleamine 2,3-dioxygenase 1; LDH, lactate dehydrogenase; LDHA, lactate dehydrogenase A; NDUFA4L2, NADH dehydrogenase 1 alpha subcomplex subunit 4-like 2; OXPHOS, oxidative phosphorylation; PDK4, pyruvate dehydrogenase kinase 4; PGC-1α, peroxisome proliferator-activated receptor gamma coactivator 1 alpha; PKM2, pyruvate kinase M2.

**Table 3:** Clinical staging and standard first-line treatment for pMMR/MSS CRC.

Stage (AJCC/UICC)	TNM	Disease Setting	Standard Treatment Strategy	Systemic Therapy	Notes
Stage 0	Tis N0 M0	Carcinoma in situ	Endoscopic resection or local excision	None	Curative local treatment; no systemic therapy
Stage I	T1–T2 N0 M0	Localized early CRC	Surgical resection	None	Surveillance; adjuvant therapy not recommended
Stage II	T3 N0 M0	Localized CRC, low-risk subgroup	Surgical resection	Surveillance or capecitabine or 5-FU/leucovorin	Observation is preferred
Stage II	T3–T4 N0 M0	Localized CRC, high-risk subgroup	Surgical resection	Surveillance or capecitabine or 5-FU/leucovorin or FOLFOX or CAPEOX	High-risk features: T4, obstruction, perforation, poor differentiation
Stage III (low- and high-risk)	Any T, N1–N2, M0	Locally advanced, node-positive CRC	Surgical resection followed by adjuvant chemotherapy	FOLFOX or CAPEOX (standard)	Adjuvant chemotherapy improves disease-free survival and OS
Stage IV (resectable synchronous liver and/or lung and metachronous metastases)	Any T, any N, M1 (limited)	Metastatic CRC with potentially curable disease	Surgery of primary tumor and metastases ± perioperative therapy	FOLFOX or CAPEOX or capecitabine or 5-FU/leucovorin	Multidisciplinary approach essential
Stage IV (unresectable synchronous liver and/or lung metastases)	Any T, any N, M1 (unresectable)	Advanced metastatic CRC	Systemic therapy (palliative intent)	FOLFIRI or FOLFOX ± panitumumab or cetuximab	*RAS/BRAF* WT and left-sided tumors
				FOLFIRI or FOLFOX or CAPEOX or FOLFIRINOX ± bevacizumab	*RAS/BRAF* mutants
Stage IV (unresectable metachronous metastases)	Any T, any N, M1 (unresectable)	Advanced metastatic CRC	Systemic therapy (palliative intent)	FOLFIRI or irinotecan ± panitumumab or cetuximab	*RAS/BRAF* WT and left-sided tumors
				FOLFIRI or irinotecan ± bevacizumab or ziv-aflibercept or ramucirumab	*RAS/BRAF* mutants
Rectal cancer (LARC)	T3–T4 and/or N+	Locally advanced rectal cancer	Total neoadjuvant therapy or chemoradiotherapy followed by surgery	Fluoropyrimidine-based chemotherapy ± oxaliplatin	Rectal cancer-specific pathway (ESMO/NCCN)

Abbreviation: CRC, colorectal cancer; FOLFOX, folinic acid, 5-FU and oxaliplatin; CAPEOX, capecitabine and oxaliplatin; FOLRFIRI, folinic acid, 5-FU and irinotecan; FOLFORINOX, folinic acid, 5-FU, irinotecan and oxaliplatin; Tis, carcinoma in situ.

**Table 4:** Clinical trials testing new therapies in CRC.

NCT	Trial Name	Phase	Status	Population/Setting	Investigational Regimen	Control	Therapeutic Response	References
NCT04262687	FFCD 1703-POCHI	II	Completed	MSS/pMMR metastatic colorectal cancer with high immune infiltrate	Pembrolizumab + CAPOX + Bevacizumab	Single-arm	NA	[179,180]
NCT04745130	sintilimab plus regorafenib phase II study	II	Completed	MSS, previously treated metastatic CRC	Sintilimab + Regorafenib	Single-arm	ORR 21.4%, DCR 63.1%, median OS 14.1 mo, median PFS 4.1 mo	[181]
NCT04362839	RIN trial	I	Completed	Chemotherapy-resistant MSS metastatic CRC	Regorafenib + Ipilimumab + Nivolumab	Single-arm	ORR ≈36.4%; median PFS ~5.0 mo; 3-yr PFS ≈19.3%; median OS ~27.5 mo	[182]
NCT03860272	C-800-01	I	Active	Advanced solid tumors; includes MSS mCRC expansion cohorts	Botensilimab ± balstilimab	Single-arm	Activity of botensilimab + balstilimab in MSS mCRC cohorts.	[183,184]
NCT05571293	NEST-1	II	Recruiting	Resectable CRC (neoadjuvant) across pMMR and dMMR cohorts	Botensilimab + balstilimab	Single-arm	Trial ongoing	NA
NCT07152821	BATTMAN	III	Not yet recruiting	Chemo-refractory, unresectable COAD	Botensilimab + balstilimab	Best supportive care (BSC)	Unavailable	NA
NCT05608044	BOT/BAL	II	Active	Refractory MSS metastatic CRC	Botensilimab ± Balstilimab	Standard of care	NA	[185]
NCT05425940	STELLAR-303	III	Active	mCRC, refractory; non-MSI-H/non-dMMR (MSS/MSI-low)	Zanzalintinib (XL092) + atezolizumab	Regorafenib	Median OS 10.9 vs. 9.4 months; HR 0.80	[186,187]
NCT04008030	CheckMate 8HW	III	Active	Unresectable or metastatic CRC with dMMR/MSI-H (immunotherapy-naïve adults; multiple lines)	Nivolumab + ipilimumab (and nivolumab monotherapy arm)	Chemotherapy	Nivolumab+ipilimumab improved outcomes vs. nivolumab alone	[188,189]
NCT02997228	COMMIT (NRG-GI004/SWOG-S1610)	III	Active	Previously untreated dMMR/MSI-H metastatic CRC	mFOLFOX6 + bevacizumab + atezolizumab	Atezolizumab monotherapy	NA	[190]
NCT02912559	ATOMIC	III	Active	Stage III colon cancer after surgery, dMMR	mFOLFOX6 + atezolizumab (then atezolizumab continuation)	mFOLFOX6	NA	[191]
NCT05723562	AZUR-1	II	Active	Treatment-naïve, locally advanced dMMR/MSI-H rectal cancer (organ preservation strategy)	Dostarlimab monotherapy	Single arm	Trial ongoing	[192]
NCT04165772	Neoadjuvant dostarlimab for dMMR/MSI tumors (includes rectal)	II	Active	Locally advanced mismatch repair-deficient/MSI solid tumors (includes rectal cancer)	Dostarlimab before standard therapy; organ-sparing approaches evaluated	Single arm	High clinical complete response and non-operative management rates reported across dMMR tumors	[193]
NCT05855200	AZUR-2	III	Recruiting	Resectable T4N0 or stage III colon cancer, dMMR/MSI-H	Perioperative dostarlimab monotherapy	Standard of care	Trial ongoing	[194]
NCT05961709	PHOENIX	II	Recruiting	Localized dMMR colon cancer (organ-sparing strategy)	Cemiplimab	Single arm	Trial ongoing	NA
NCT06959550	Ivonescimab (anti-PD-1/VEGF bispecific) in mCRC	II	Recruiting	Previously treated mCRC; cohorts include dMMR/MSI-H post-anti-PD-1, MSS with/without liver mets	Ivonescimab	Single arm	Trial ongoing	NA
NCT05217446	SEAMARK	II	Active	Previously untreated metastatic CRC with BRAF V600E and MSI-H/dMMR	Encorafenib + cetuximab + pembrolizumab	Pembrolizumab	Trial ongoing	[195]
NCT04607421	BREAKWATER	III	Active	Previously untreated BRAF V600E-mutant mCRC	Encorafenib + cetuximab + mFOLFOX6 (and other EC ± chemo arms)	Chemotherapy ± bevacizumab	ORR improved with EC+mFOLFOX6 vs. control	[196,197]
NCT04017650	ECN trial	I/II	Active	MSS BRAF V600E metastatic CRC	Encorafenib + cetuximab + nivolumab	Single arm	ORR ~50%; median PFS ~7.4 mo	[198]
NCT03388190	METIMMOX	II	Completed	First-line MSS metastatic CRC (including BRAF V600E)	Encorafenib + cetuximab + nivolumab ± chemotherapy	Targeted + immunotherapy alone	ORR & PFS higher in chemo-containing arm; improved outcomes vs. non-chemo arm	[199]
NCT05198934	CodeBreaK 300	III	Active	Chemorefractory KRAS G12C-mutated mCRC	Sotorasib + panitumumab	Investigator’s choice (trifluridine–tipiracil or regorafenib)	PFS improved vs. standard therapies	[200,201,202]
NCT04793958	KRYSTAL-10	III	Active	Second-line KRAS G12C-mutated mCRC	Adagrasib + cetuximab	Chemotherapy (FOLFIRI or mFOLFOX6)	Trial ongoing	NA
NCT03785249	KRYSTAL-1	I/II	Active	KRAS G12C-mutated advanced solid tumors; includes mCRC cohorts	Adagrasib ± cetuximab	Single arm cohorts	Antitumor activity in heavily pretreated KRAS G12C mCRC	[203]
NCT04449874	GO42144 (divarasib ± combinations)	I	Recruiting	KRAS G12C-positive advanced solid tumors; includes CRC cohorts (divarasib + cetuximab arm)	Divarasib (GDC-6036) + cetuximab (CRC cohort) and other combinations	Single arm	Divarasib + cetuximab showed encouraging activity in KRAS G12C mutated CRC patients	[204]
NCT07020221	VS-7375 (KRAS G12D inhibitor)	I/II	Recruiting	Advanced solid tumors with KRAS G12D; includes CRC KRAS G12D expansion with cetuximab	VS-7375 ± cetuximab (CRC cohort)	Single arm	Trial ongoing	NA
NCT06917079	BBO-11818 (pan-KRAS inhibitor)	I	Recruiting	KRAS-mutant advanced solid tumors (CRC eligible)	BBO-11818 (alone and combinations per protocol)	Single-arm dose-escalation/expansion	Trial ongoing	NA
NCT03043313	MOUNTAINEER	II	Completed	First-line HER2+ metastatic CRC (RAS wild-type)	Tucatinib + trastuzumab	Single arm	ORR: 39.3%; median PFS: 8.1 mo; median OS: 23.9 mo	[205]
NCT05253651	MOUNTAINEER-03	III	Recruiting	First-line HER2+ metastatic CRC (RAS wild-type)	Tucatinib + trastuzumab + mFOLFOX6	mFOLFOX6 ± bevacizumab or cetuximab (SOC)	Trial ongoing	[11]
NCT04744831	DESTINY-CRC02	II	Completed	HER2-overexpressing metastatic CRC	Trastuzumab deruxtecan	Single arm	ORR ~45–46%; DCR ~75–80%; median PFS ~7–9.3 mo	[206]
NCT06243393	TROPHIT1	II/III	Recruiting	Metastatic CRC refractory to ≥2 lines of SOC	Sacituzumab govitecan (SG)	Standard of care	Trial ongoing	[207]
NCT05379595	OrigAMI-1	I/II	Recruiting	Advanced/metastatic CRC; cohorts include RAS/BRAF WT (e.g., rechallenge/sidedness cohorts)	Amivantamab monotherapy and amivantamab + SOC chemotherapy cohorts	Non-randomized (multiple cohorts)	Trial ongoing	NA
NCT06750094	OrigAMI-3	III	Recruiting	KRAS/NRAS & BRAF wild-type recurrent/unresectable/mCRC after prior chemotherapy	Amivantamab + FOLFIRI	Cetuximab or bevacizumab + FOLFIRI	Trial ongoing	NA
NCT07023289	ABBV-400 ctDNA+ CRC (post-adjuvant NED)	II	Recruiting	Post-adjuvant ctDNA-positive CRC with no radiographic evidence of disease (NED)	Telisotuzumab adizutecan (ABBV-400) monotherapy	Standard of care	Trial ongoing	NA
NCT04929223	INTRINSIC (umbrella)	I	Recruiting	Biomarker-selected metastatic CRC (umbrella arms)	Targeted therapies/immunotherapy (arm-specific; includes divarasib combos)	Single arm	Trial ongoing	NA

Abbreviations: NA, not available; ORR, Objective response rate; PFS = Progression-free survival.

## Data Availability

No new data were created or analyzed in this study.

## Supplementary Materials

The following supporting information can be downloaded at: https://www.mdpi.com/article/10.3390/cells15121074/s1, Table S1: Clinical staging and standard treatment for dMMR/MSI-H colon cancer; Table S2: Second line treatment for advanced or metastatic CRC following NCCN guidelines.

## Abbreviations

The following abbreviations are used in this manuscript:

5-FU	5-Fluorouracil
ACSL4	Acyl-CoA synthetase long-chain family member 4
AKT	Protein kinase B
AMPK	AMP-activated protein kinase
APC	Adenomatous polyposis coli
ASs	Artificial sweeteners
ATG	Autophagy-related gene
BECN1	Beclin-1
BHB	Beta-hydroxybutyrate
BMI	Body mass index
BPA	Bisphenol A
BRAF	B-Rapidly Accelerated Fibrosarcoma
CAA	Cancer-associated adipocyte
CAPEOX	Capecitabine plus oxaliplatin
CD36	Cluster of differentiation 36
CMS	Consensus molecular subtype
COAD	Colon adenocarcinoma
CPT1A	Carnitine palmitoyltransferase 1A
CRC	Colorectal cancer
CS	Citrate synthase
CTLA-4	Cytotoxic T-lymphocyte-associated protein 4
DC	Dendritic cell
dMMR	Deficient mismatch repair
EGFR	Epidermal growth factor receptor
EMT	Epithelial–mesenchymal transition
EOCRC	Early-onset colorectal cancer
ER	Estrogen receptor
ERK	Extracellular signal-regulated kinase
FA	Fatty acid
FAO	Fatty acid oxidation
FASN	Fatty acid synthase
FH	Fumarate hydratase
FOXP3	Forkhead box P3
FOLFIRI	Folinic acid, fluorouracil and irinotecan
FOLFOX	Folinic acid, fluorouracil and oxaliplatin
FOLFOXIRI	Folinic acid, fluorouracil, irinotecan and oxaliplatin
FXR	Farnesoid X receptor
GCGR	Glucagon receptor
GEO	Gene Expression Omnibus
GLP-1	Glucagon-like peptide-1
GLP-1RA	Glucagon-like peptide-1 receptor agonist
GLUT1	Glucose transporter 1
GMRGs	Glutamine metabolism-related genes
GPER	G protein-coupled estrogen receptor
HDAC	Histone deacetylase
HER2	Human epidermal growth factor receptor 2
HIF-1α	Hypoxia-inducible factor 1 alpha
HK2	Hexokinase 2
HLA	Human leukocyte antigen
HMW	High molecular weight
ICB	Immune checkpoint blockade
ICD	Immunogenic cell death
ICI	Immune checkpoint inhibitor
IDO	Indoleamine 2,3-dioxygenase
IDH	Isocitrate dehydrogenase
IFN-γ	Interferon gamma
IGF	Insulin-like growth factor
IGFBP	Insulin-like growth factor binding protein
IHC	Immunohistochemistry
IL	Interleukin
JAK	Janus kinase
KRAS	Kirsten rat sarcoma viral oncogene homolog
LAMP	Lysosome-associated membrane protein
LAT1	L-type amino acid transporter 1
LDH	Lactate dehydrogenase
LPCAT1	Lysophosphatidylcholine acyltransferase 1
LPS	Lipopolysaccharide
mAb	Monoclonal antibody
MAP1LC3	Microtubule-associated protein 1 light chain 3
MAPK	Mitogen-activated protein kinase
mCRC	Metastatic colorectal cancer
MDSC	Myeloid-derived suppressor cell
MMR	Mismatch repair
mTOR	Mechanistic target of rapamycin
MSI	Microsatellite instability
MSI-H	Microsatellite instability-high
MSS	Microsatellite stable
NCCN	National Comprehensive Cancer Network
NDUFA4L2	NADH dehydrogenase 1 alpha subcomplex subunit 4-like 2
NF-κB	Nuclear factor kappa B
NK	Natural killer
NLRP3	NLR family pyrin domain containing 3
NO	nitric oxide
ORR	Objective response rate
OS	Overall survival
OXPHOS	Oxidative phosphorylation
PAK1	P21 (RAC1) activated kinase 1
PD-1	Programmed cell death protein 1
PD-L1	Programmed death ligand 1
PDK	Pyruvate dehydrogenase kinase
PFS	Progression-free survival
PGC-1α	Peroxisome proliferator-activated receptor gamma coactivator 1 alpha
PHGDH	Phosphoglycerate dehydrogenase
PI3K	Phosphoinositide 3-kinase
PKM2	Pyruvate kinase M2
POLE	Polymerase epsilon
PSAT1	Phosphoserine aminotransferase 1
PSPH	Phosphoserine phosphatase
RAS	Rat Sarcoma
ROS	Reactive oxygen species
SCFAs	Short-chain fatty acids
SDH	Succinate dehydrogenase
SGOC	Serine–glycine one-carbon metabolism
SIRT1	Sirtuin 1
SLC2A1	Solute carrier family 2 member 1
STAT3	Signal transducer and activator of transcription 3
T2DM	Type 2 diabetes mellitus
TAM	Tumor-associated macrophage
TCA	Tricarboxylic acid cycle
TDO	tryptophan 2,3-dioxygenase
TCGA	The Cancer Genome Atlas
TGF-β	Transforming growth factor beta
Th	T helper
TIL	Tumor infiltrating lymphocyte
TLS	Tertiary lymphoid structure
TLR4	Toll-like receptor 4
TMB	Tumor mutational burden
TME	Tumor microenvironment
TNF-α	Tumor Necrosis Factor alpha
Treg	Regulatory T cell
UPF	Ultra-processed food
VAT	Visceral adipose tissue
VEGF	Vascular endothelial growth factor
VEGFR	Vascular endothelial growth factor receptor
VFA	Visceral fat area
WHR	Waist-to-hip ratio
