# Supplementary material for: Determinants of Colorectal Cancer: An Integrative Immunometabolic Framework Linking Biomarkers, Therapy, and the Diet–Microbiota Axis
Source: Cells. 2026 Jun 13;15(12):1074. doi: 10.3390/cells15121074 (PMC13297663; doi:10.3390/cells15121074)
Supplement: Supplementary file 1 [file cells-15-01074-s001.zip › cells-4289441-supplementary/Supplementary Table S1.pdf]

Supplementary Table S1. Clinical staging and standard treatment for dMMR/MSI-H colon cancer.

Stage (AJCC/UICC)	TNM	Disease setting	Standard treatment strategy	Systemic therapy	Notes
Stage 0	Tis N0 M0	Carcinoma in situ	Endoscopic resection or local excision	None	Curative local treatment; no systemic therapy
Stage I	T1–T2 N0 M0	Localized early CRC	Surgical resection	None	Surveillance; adjuvant therapy not recommended
Stage II	T3-T4a N0 M0	Localized CRC	Surgical resection	Surveillance	Observation is preferred
Stage II	T4b N0 M0	Localized CRC	Surgical resection	FOLFOX or CAPEOX ± atezolizumab	immunotherapy
Stage III (low- and high-risk)	Any T, N1–N2, M0	Locally advanced, node-positive CRC	Surgical resection followed by adjuvant chemotherapy		
Stage IV (resectable synchronous liver and/or lung or metachronous metastases)	Any T, any N, M1	Metastatic CRC with potentially curable disease	Surgery of primary tumor and metastases	FOLFOX or CAPEOX or capecitabine or 5-FU/leucovorin	Multidisciplinary approach essential
			Ceckpoint inhibitor immunotherapy followed by surgery of primary tumor and metastases	Surveillance	
Stage IV (synchronous abdominal/peritoneal metastases)	Any T, any N, M1	Metastatic CRC with potentially curable disease	If obstructed, colon resection or other surgical treatments	Ceckpoint inhibitor immunotherapy	Evaluation of disease status after 2-3 months

Stage IV (unresectable synchronous metastases)	Any T, any N, M1 (unresectable)	Advanced metastatic CRC	Systemic therapy (palliative intent)	Ceckpoint inhibitor immunotherapy	Evaluation of disease status after 2-3 months

Tis: carcinoma in situ. CRC: colorectal cancer; FOLFOX: folinic acid, fluorouracil (5-FU) and oxaliplatin; CAPEOX: capecitabine and oxaliplatin; FOLRFIRI: folinic acid, fluorouracil (5-FU) and irinotecan; FOLFORINOX: folinic acid, fluorouracil (5FU), irinotecan and oxaliplatin.
