# Supplementary material for: Determinants of Colorectal Cancer: An Integrative Immunometabolic Framework Linking Biomarkers, Therapy, and the Diet–Microbiota Axis
Source: Cells. 2026 Jun 13;15(12):1074. doi: 10.3390/cells15121074 (PMC13297663; doi:10.3390/cells15121074)
Supplement: Supplementary file 1 [file cells-15-01074-s001.zip › cells-4289441-supplementary/Supplementary Table S2.pdf]

Supplementary Table S2. Second line treatment for advanced or metastatic CRC following NCCN guidelines.

Clinical Setting	Molecular Profile	Second-Line Recommended Treatment	Strength / Notes
mCRC treated with oxaliplatin-based first line therapy without irinotecan	RAS/BRAF wild-type	FOLFIRI + anti-EGFR (cetuximab or panitumumab); cetuximab or panitumumab ± irinotecan	preferred option; higher response rate
	RAS/BRAF mutants	FOLFIRI or irinotecan ± bevacizumab or aflibercept or ramucirumab	anti-angiogenic therapy recommended
mCRC treated with irinotecan-based first line therapy without oxaliplatin	RAS/BRAF wild-type	FOLFOX or CAPEOX + cetuximab or panitumumab; cetuximab or panitumumab ± irinotecan	switch chemotherapy backbone
	RAS/BRAF mutants	FOLFOX or CAPEOX ± bevacizumab	anti-angiogenic therapy recommended
mCRC treated with first line therapy without oxaliplatin and irinotecan	RAS/BRAF wild-type	FOLFIRI + cetuximab or panitumumab; cetuximab or panitumumab ± irinotecan	preferred option
	RAS/BRAF mutants	FOLFOX or CAPEOX ± bevacizumab; FOLFIRI or irinotecan ± bevacizumab or aflibercept or ramucirumab; irinotecan + oxaliplatin ± bevacizumab; FOLFIRINOX ± bevacizumab	anti-angiogenic therapy recommended

mCRC treated with first line therapy with oxaliplatin and irinotecan	RAS/BRAF wild-type	cetuximab or panitumumab ± irinotecan	preferred option
mCRC, previously treated	BRAF V600E mutation	encorafenib + cetuximab/panitumumab	standard of care after first line
mCRC, previously treated	HER2 amplified, RAS/BRAF wild-type	trastuzumab combinations with pertuzumab or tucatinib or lapatinib	after failure of standard chemotherapy
mCRC, previously treated	NTRK gene fusion	entrectinib; larotrectinib; repotrectinib	after failure of standard chemotherapy
mCRC, previously treated with systemic therapy	dMMR/MSI-H	ceckpoint inhibitor immunotherapy	immunotherapy recommended
mCRC, previously treated with immunotherapy	dMMR/MSI-H	systemic therapy or nivolumab + ipilimumab	if ceckpoint inhibitor monotherapy was previously administrated

Tis: carcinoma in situ. CRC: colorectal cancer; FOLFOX: folinic acid, fluorouracil (5-FU) and oxaliplatin; CAPEOX: capecitabine and oxaliplatin; FOLRFIRI: folinic acid, fluorouracil (5-FU) and irinotecan; FOLFORINOX: folinic acid, fluorouracil (5FU), irinotecan and oxaliplatin.
